# Enhancing response of a protein conformational switch by using two disordered ligand binding domains

**DOI:** 10.3389/fmolb.2023.1114756

**Published:** 2023-03-02

**Authors:** Harsimranjit Sekhon, Jeung-Hoi Ha, Stewart N. Loh

**Affiliations:** Department of Biochemistry and Molecular Biology, SUNY Upstate Medical University, Syracuse, NY, United States

**Keywords:** alternate frame folding, mutually exclusive folding, loop closure entropy, allostery, protein engineering

## Abstract

**Introduction:** Protein conformational switches are often constructed by fusing an input domain, which recognizes a target ligand, to an output domain that establishes a biological response. Prior designs have employed binding-induced folding of the input domain to drive a conformational change in the output domain. Adding a second input domain can in principle harvest additional binding energy for performing useful work. It is not obvious, however, how to fuse two binding domains to a single output domain such that folding of both binding domains combine to effect conformational change in the output domain.

**Methods:** Here, we converted the ribonuclease barnase (Bn) to a switchable enzyme by duplicating a C-terminal portion of its sequence and appending it to its N-terminus, thereby establishing a native fold (OFF state) and a circularly permuted fold (ON state) that competed for the shared core in a mutually exclusive fashion. Two copies of FK506 binding protein (FKBP), both made unstable by the V24A mutation and one that had been circularly permuted, were inserted into the engineered barnase at the junctions between the shared and duplicated sequences.

**Results:** Rapamycin-induced folding of FK506 binding protein stretched and unfolded the native fold of barnase *via* the mutually exclusive folding effect, and rapamycin-induced folding of permuted FK506 binding protein stabilized the permuted fold of barnase by the loop-closure entropy principle. These folding events complemented each other to turn on RNase function. The cytotoxic switching mechanism was validated in yeast and human cells, and *in vitro* with purified protein.

**Discussion:** Thermodynamic modeling and experimental results revealed that the dual action of loop-closure entropy and mutually exclusive folding is analogous to an engine transmission in which loop-closure entropy acts as the low gear, providing efficient switching at low ligand concentrations, and mutually exclusive folding acts as the high gear to allow the switch to reach its maximum response at high ligand concentrations.

## Introduction

Engineering allosteric mechanisms into natural proteins has enabled the development of protein switches with many applications in synthetic biology. A common means to accomplish this goal is to develop mechanisms for coupling an input domain (which is responsible for recognizing a target ligand or other stimulus) to an output domain (which provides biological activity) by means of protein conformational change. This challenge has been addressed by methods that split a protein into two fragments and reconstitute them in the presence of a ligand (Zetsche et al., 2015; Diaz et al., 2017; Dagliyan et al., 2018), induce a domain swap upon ligand binding (Ha et al., 2015; Karchin et al., 2017), and cause a conformational change in the protein in response to a stimulus (Mitrea et al., 2010; Stratton and Loh, 2011; Dagliyan et al., 2016; Guo et al., 2022). Here, we combine three protein engineering strategies—alternate frame folding, mutually exclusive folding, and loop enclosure entropy—to develop a conformation-switching enzyme that activates upon the binding of rapamycin or FK506.

**FIGURE 1:**
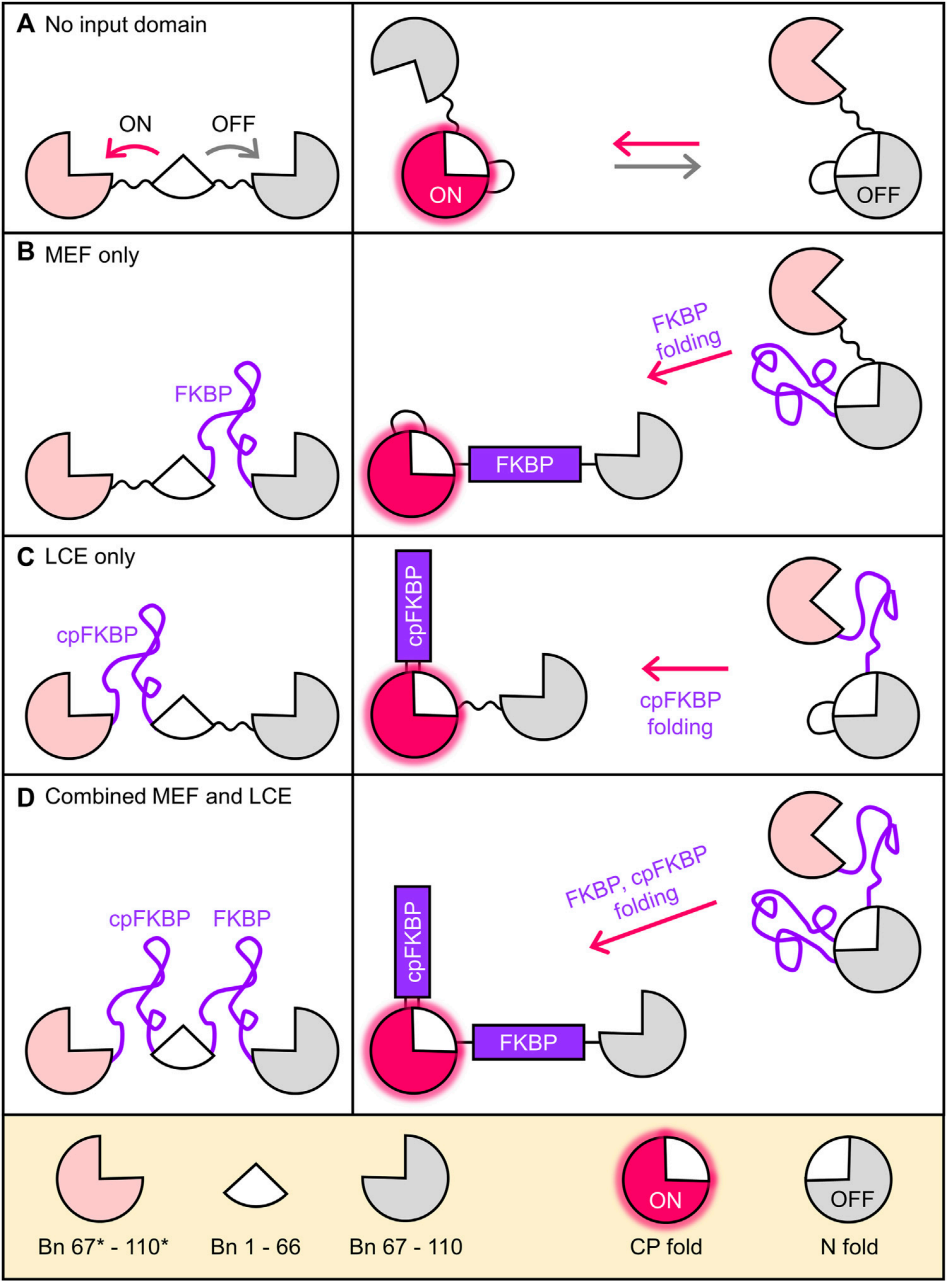
Coupling folding of FKBP receptor domains to the Bn-AFF fold shift. **(A)** By duplicating a portion of the Bn sequence, Bn-AFF switches between the N-fold (OFF state) and the CP-fold (ON state). Mutual exclusivity of folding is established by competition of the two duplicate segments (pink and grey) for the same shared segment (white). Without an FKBP receptor domain this occurs in a spontaneous, unregulated fashion. **(B)** To simulate MEF switching, an unstable FKBP domain was inserted into the N-frame at the surface loop separating the shared and duplicate regions. Folding of FKBP splits apart the N-fold and the switch shifts to the CP-fold. **(C)** LCE switching was simulated by placing an unstable cpFKBP domain between the duplicate and shared regions of the CP-fold. cpFKBP folding removes the entropic penalty to folding of the CP-fold and drives the N-to-CP conformational change. **(D)** The full MEF-LCE switch was constructed by inserting both FKBP and cpFKBP at the points described above. Folding of these domains provides maximum driving force for Bn-AFF activation.

Alternate frame folding (AFF) introduces allosteric control into a protein of interest (POI) such that its activity can be switched off and on by an external stimulus such as ligand binding (Stratton et al., 2008; Stratton and Loh, 2011; Do and Boxer, 2013; Ni et al., 2019; Gräwe and Merkx, 2022). The POI is first conceptually bisected into two semi-arbitrarily chosen fragments, with the main consideration being the split site should be at a surface loop in the POI. The fragment that contains a critical functional residue of choice is then duplicated and appended to the terminus of the other fragment using a flexible linker. This process is illustrated in Figure 1A using the ribonuclease barnase (Bn; 110 amino acids). The 67–110 fragment (gray; containing the general acid His102) is copied and pasted to the N-terminus of Bn, with the new sequence (67*–110*, pink) denoted by asterisks. The resulting molecule (Bn-AFF) switches between the native fold (N-fold) and a circularly permuted fold (CP-fold) defined so because Bn is in circularly permuted form (cpBn). Mutual exclusivity of folding is established by the competition of the pink and grey fragments for the white fragment (residues 1–66). Mutating His102 to Ala in the gray sequence but not in the pink sequence establishes the N-fold as the OFF state and the CP-fold as the ON state.

The above steps convert Bn to a conformational switch capable of ON/OFF RNase output, but without an input domain Bn-AFF is unregulated (Figure 1A). We previously introduced two mechanisms by which folding of an input domain can be used to control the AFF-mediated conformational change. The first method, termed mutually exclusive folding (MEF) (Radley et al., 2003; Cutler and Loh, 2007; Cutler et al., 2009), inserts a recognition domain [FK506 binding protein (FKBP)] into the N-fold in between the duplicated and shared segments (Figure 1B). FKBP was previously made unstable in the absence of its cognate ligand (FK506 or rapamycin) by the V24A mutation (Fulton et al., 1999), and in this disordered state it simply extends the surface loop that is centered around residue 66 when the protein is in the N-fold. FK506 or rapamycin binding induces FKBP to fold, causing its N-to-C termini to separate to the native distance of 27 Å. The native FKBP structure then stretches apart the N-fold, allowing Bn-AFF to shift into the CP-fold (Figure 1B).

The second regulatory mechanism, named entropy switching (John et al., 2022), is based on the principle of loop-closure entropy (Minard et al., 2001; Scalley-Kim et al., 2003) (LCE). A circular permutant of the same unstable FKBP domain as above (cpFKBP) is inserted in between the shared and duplicated segments of the CP-fold of Bn-AFF (Figure 1C), where it acts as a surface loop in the CP-fold. When cpFKBP is disordered, it introduces a thermodynamic penalty to the folding of the CP-fold that arises from the entropic cost of constraining the ends of the flexible cpFKBP polypeptide to a single distance. This penalty is theoretically proportional to the logarithm of number of cpFKBP disordered residues. The disorder-to-order transition, induced by rapamycin binding, removes the LCE penalty, stabilizes the CP-fold, and drives the OFF to ON transition.

In this work we used dual FKBP domains to trigger the Bn-AFF fold shift by the combined actions of MEF and LCE. RNase activation by rapamycin binding was demonstrated by enzymatic assays *in vitro*, protein expression knockdown in mammalian cells, and *S. cerevisiae* cell toxicity. We presented a thermodynamic model that describes the coupled conformational changes in this class of dual-action switch. To our knowledge, Bn-AFF is the first example of a switch in which two essentially identical input domains were employed to amplify the output response. The engineering approach described herein can be applied to other input and output domains to create switches with enhanced response.

## Results

### Simulating combined MEF and LCE switching

To illustrate how Bn-AFF molecules flow from OFF to ON states in response to FKBP/cpFKBP domain folding (induced by rapamycin binding), we developed a thermodynamic model that describes the interconversion between all states of the switch. Assuming two-state character for all folding/unfolding reactions, the Bn domain of Bn-AFF can exist in three conformations (N-fold, CP-fold, or globally unfolded), and the FKBP and cpFKBP domains can each exist in folded or unfolded conformations. The minimal structural model of Bn-AFF switching therefore consists of 3 × 2 × 2 = 12 states. Ligand was allowed to bind to the states in which the FKBP and/or cpFKBP domains were folded, with the same K_d_ value of 1 nM. The free energies of FKBP and cpFKBP unfolding were set to −2 kcal/mol in all simulations, making 97% of those domains unfolded in the absence of rapamycin. Without ligand, most Bn-AFF molecules were kept in the OFF state by making the N-fold more stable than the CP-fold by variable amounts. The fraction of molecules in the ON state was calculated by the number of molecules in the CP-fold divided by the total number of molecules.

The main goals of the simulations were to quantify how MEF and LCE individually cause the molecules to flow from N-fold (OFF) to CP-fold (ON) states as a function of added ligand, and how combining MEF and LCE provides added benefits. Accordingly, we first simulated the MEF-only switch using a simplified model (6 states) generated from a construct with only FKBP in the N-frame and no cpFKBP in the CP-frame (Figure 1B; Supplementary Figure S1). We then simulated the LCE-only switch (6 states) using the complementary construct that contained only cpFKBP in the CP-frame (Figure 1C; Supplementary Figure S2). Finally, we combined the two to generate the simulation of the complete 12-state Bn-AFF switch (Figure 1D; Supplementary Figure S3). Full details of the models and simulations can be found in Supplementary Note S2.

**FIGURE 2:**
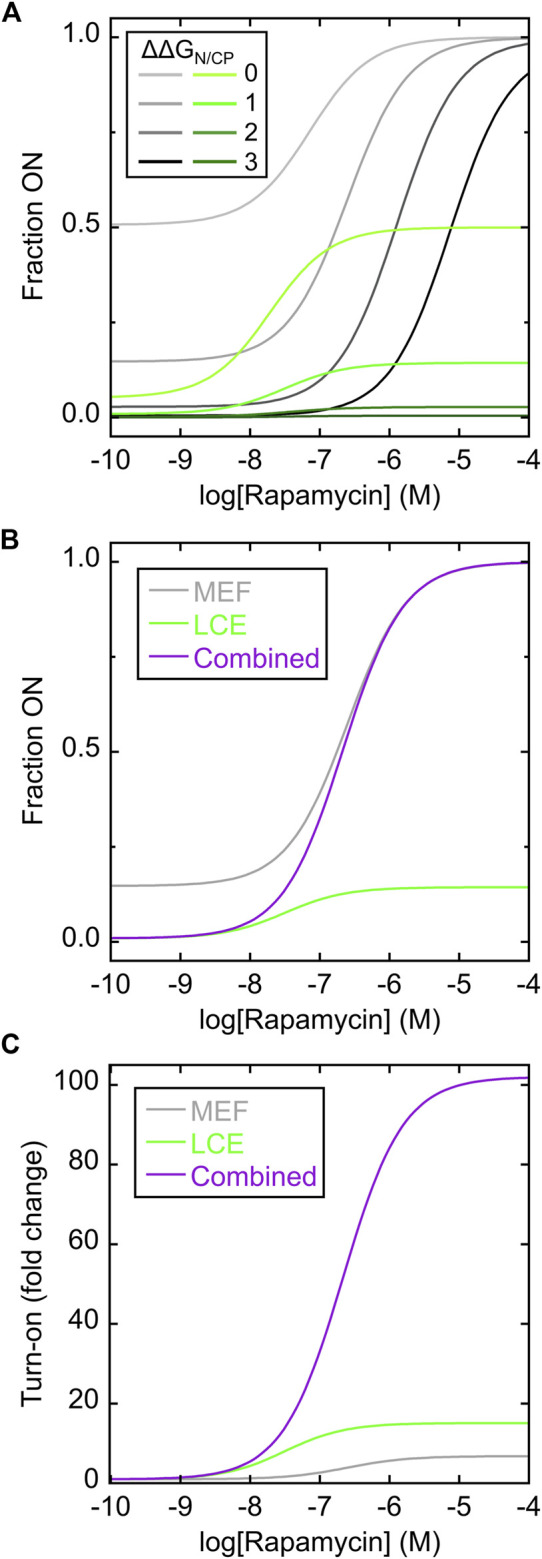
Simulating MEF-only, LCE-only, and MEF-LCE switching mechanisms. **(A)** Rapamycin response curves of the MEF-only switch (grey lines; c.f. Figure 1B) and the LCE-only switch (green lines; c.f. Figure 1C) were simulated using the values of ΔΔG_N/CP_ indicated in the inset (in units of kcal/mol). K_d_ of rapamycin binding to folded FKBP and cpFKBP domains was set to 1 nM. The LCE penalty was set to −2 kcal/mol. **(B)** The simulated response curve of the MEF-LCE switch (purple; c.f. Figure 1D) combined the low background activity of LCE-only switching (green) with the high activity ceiling of MEF-only switching (grey). The ΔΔG_N/CP_ value was set to 1 kcal/mol and all other parameters were identical to **(A)**. **(C)** The dual MEF-LCE switch exhibited a theoretical ∼100-fold turn on of activity in the conditions of **(B)**, equating to 15.1-fold and 6.7-fold increases over that of MEF-alone and LCE-alone, respectively. Full description of the models (Supplementary Figures S1–S3) and populations of all significantly occupied states in each model (Supplementary Figure S4) are included in Supplementary Information.

The MEF condition was established as previously described (Cutler et al., 2009; Woloschuk et al., 2021) by disallowing any state in which the FKBP domain and the N-fold (of the Bn domain) were both folded. Simulated rapamycin dose-response curves were plotted as a function of the difference in unfolding free energies of the N-fold and CP-fold (ΔΔG_N/CP_, where positive values indicate the N-fold is more stable than the CP-fold). ΔΔG_N/CP_ defines the extent to which the switch is OFF in the absence of rapamycin and thus determines the maximum signal change of the switch. For example, when ΔΔG_N/CP_ equals zero, the switch exists almost entirely as a 50/50 mix of N-fold and CP-fold (Figure 2A; Supplementary Figure S4A) and the maximum turn-on is a factor of two. The MEF-only simulation revealed that rapamycin addition converted the single-FKBP switch from a mostly OFF state to the fully ON state (Figure 2A; gray curves). The shapes of the response curves were that of the standard one-site binding isotherm. The apparent K_d_ values for activation became progressively higher than the canonical K_d_ (1 nM) as ΔΔG_N/CP_ increased, reflecting the energetic cost to push the balance from the N-fold to the increasingly destabilized CP-fold. This cost is paid by ligand binding energy. This relationship illustrates the trade-off between maximizing the signal change (high turn-on, enforced by large ΔΔG_N/CP_ values), and the resulting need for large concentrations of ligand to activate the switch.

The LCE condition was established by introducing an energetic penalty to folding of the CP-fold when the inserted cpFKBP domain is unfolded. The LCE penalty can be experimentally determined for a given protein pair, but technical considerations prevented us from doing so for the present system (*vide infra*). We estimated the LCE penalty to be −2 kcal/mol in the simulations. The simulated rapamycin response curves revealed that LCE alone activated switching (Figure 2A; green curves), but with three differences compared to MEF alone (Figure 2A; gray curves). First, the apparent K_d_ of rapamycin binding in the LCE model is lower than that in the MEF model at any given value of ΔΔG_N/CP_, meaning LCE activates the switch more effectively than MEF at low ligand concentrations. Second, compared to MEF, LCE results in more molecules being OFF in the absence of ligand thus establishing a lower background signal. At high ligand concentrations, however, the LCE effect saturates and LCE may not fully drive conversion to the ON state. By contrast, MEF will always convert all molecules to the ON state, given high enough ligand concentration. In this respect, LCE and MEF are complementary.

Figure 2B superimposes the responses of the MEF-only and LCE-only switches with that of the combined MEF-LCE switch (containing FKBP in the N-fold and cpFKBP in the CP-fold) in the case where ΔΔG_N/CP_ = 1 kcal/mol. The MEF-LCE switch combines the low background signal of LCE with the high ceiling of the MEF response. The net result is an increase in turn-on of the dual switch at all ligand concentrations, where turn-on is defined as the fraction of molecules in the CP-fold at a given [rapamycin] divided by the fraction of molecules in the CP-fold with zero rapamycin (Figure 2C). The combined switch affords as much as 15.1-fold and 6.7-fold improvements over MEF alone and LCE alone, respectively, in the conditions of Figure 2C. In summary, the simulations demonstrate that introducing two receptor domains can significantly enhance the AFF-mediated switching response, through the combined action of MEF and LCE.

### Characterization of switch components

**FIGURE 3:**
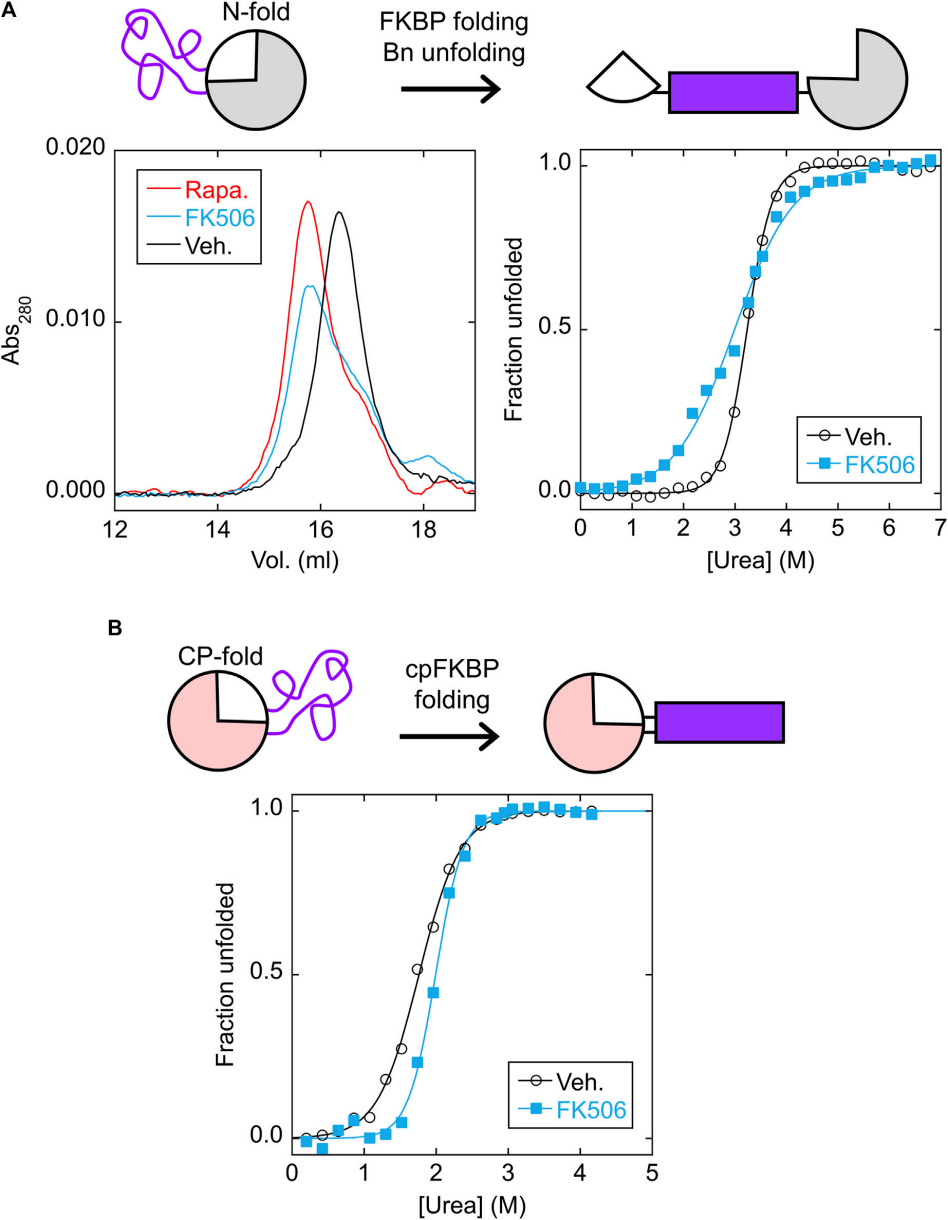
FKBP folding destabilizes the N-fold analog *via* MEF, and cpFKBP folding stabilizes the CP-fold analog *via* LCE. **(A)** SEC chromatograms (left) found that the N-fold analog eluted at a higher apparent MW in the presence of rapamycin or FK506, suggesting that binding to the FKBP domain destabilized and at least partially unfolded the Bn domain. ΔG_unfold_ and C_m_ values (Supplementary Table S1) obtained from urea denaturation curves (right) supported this conclusion. **(B)** Urea denaturation curves of the CP-fold analog found that FK506 binding increased ΔG_unfold_ and C_m_ (Supplementary Table S1), consistent with removal of the loop-closure entropy penalty imposed by the unfolded cpFKBP domain. Each point is an average of three technical repeats. Raw data are plotted in Supplementary Figure S4.

We purified the isolated N-frame analog (Bn with FKBP inserted between residues 66–67) and CP-frame analog (cpBn with cpFKBP inserted between residues 110* and 1) to ascertain the effects of FKBP/cpFKBP insertion on Bn/cpBn stability, vis-à-vis the MEF and LCE mechanisms, respectively. The N-frame analog is expected to consist of folded Bn with an extended, disordered surface loop composed of the cpFKBP sequence (Figure 3A, top schematic). Upon addition of rapamycin or FK506, MEF predicts that the FKBP domain will fold and destabilize the Bn domain, potentially unfolding the latter and splitting it into two fragments connected by FKBP. As a test, we mixed the N-frame analog (24.6 kDa) with rapamycin, FK506, or DMSO vehicle and analyzed the proteins by size exclusion chromatography (SEC). The rapamycin and FK506 samples eluted at the same volume (apparent MW of 48 kDa) and earlier than the vehicle control (apparent MW of 33 kDa; Figure 3A, left). The observation that the apparent MW increased by less than a factor of two suggested that FKBP folding caused Bn to at least partially unfold, but not go on to form a domain-swapped dimer in which all domains are folded, a phenomenon that we reported earlier in other MEF systems (Ha et al., 2015; Ha et al., 2012; Karchin et al., 2017).

A unique signature of MEF is the seemingly paradoxical effect of the host protein becoming destabilized in the presence of ligand. Thus, as a further test we performed urea denaturation experiments on the N-frame analog in the presence and absence of FK506 using Trp fluorescence to monitor unfolding (Figure 3A, right). Since three of the four Trp residues are in the Bn domain, we expected that fluorescence would report mostly on Bn conformation. As expected, the N-frame analog appeared to be much less stable with FK506 (ΔG_unfold_ = 3.2 ± 0.1 kcal/mol) than without the drug (ΔG_unfold_ = 7.6 ± 0.7 kcal/mol) (Supplementary Figure S5A; Supplementary Table S1). Related to the drop in ΔG_unfold_, another indication of MEF is the broadening of the Bn unfolding transition (demonstrated by a decrease in the cooperativity parameter *m*) in the presence of ligand. A decrease in *m* is normally interpreted as the presence of additional, poorly-resolved unfolding transition(s) caused by accumulation of one or more unfolding intermediates. In the case of MEF, however, *m* decreases due to the simultaneous unfolding of the host domain and folding of the inserted domain even as the overall transition remains two-state with no populated intermediate (Cutler et al., 2009). The observed *m*-value of Bn reduces to the difference in *m*-values of the Bn and FKBP domains under ideal experimental conditions, i.e., when Bn cannot fold when FKBP is folded, and when FKBP remains stable at denaturant concentrations that unfold Bn. These conditions were not rigorously established in the present case. Nevertheless, the decrease in *m* and ΔG_unfold_ provides evidence that FK506 binding destabilizes the N-fold as predicted by MEF.

We next asked whether the CP-frame analog was stabilized by binding-induced folding of the cpFKBP domain as predicted by LCE (Figure 3B, top schematic). In agreement, adding rapamycin increased the fitted ΔG_unfold_ from 3.3 ± 0.1 kcal/mol to 5.7 ± 0.7 kcal/mol and C_m_ from 1.76 ± 0.01 M to 1.99 ± 0.06 M (Supplementary Figure S5B; Supplementary Table S1). The difference in ΔG_unfold_ (2.4 kcal/mol) theoretically equals the LCE penalty. Because cpFKBP unfolding may have contributed to the observed fluorescence change (due to the Trp residue in the cpFKBP domain), this equivalency was not proven. However, the observation that both ΔG_unfold_ and C_m_ increased with rapamycin addition strongly implies that the Bn domain was stabilized by rapamycin according to the LCE model.

### Tuning the Bn-AFF switch

A key step in constructing an AFF-based switch is tuning the thermodynamic balance between the N- and CP-frames. This process generates the ΔΔG_N/CP_ values depicted in the simulations of Figure 2A and determines the extent to which the switch is off in the absence of drug and how much ligand is needed to turn it on. The desired balance will depend on the application, which in our case is to destroy endogenous RNA in cells treated with rapamycin. We therefore tuned Bn-AFF in HEK293T cells using mRNA degradation as the reporter. Cells were transfected with plasmids expressing Bn-AFF with Ile (the WT residue), Val, Ala, and Gly at position 96* in the CP-frame (Wood et al., 2018). I96 is buried in the hydrophobic core of Bn and replacing Ile with smaller side chains was predicted to progressively destabilize the CP-fold, causing the switch to adopt the catalytically inactive N-fold and reducing background activity. A reporter plasmid expressing mCherry, tagged with a PEST sequence to reduce protein half-life, was co-expressed to provide fluorescence readout *via* mCherry mRNA degradation. The control was Bn-AFF made fully inactive by introducing the H102*A catalytic mutation into the CP-frame.

Transfecting cells with the H102*A control resulted in many brightly fluorescent cells, as expected (Supplementary Figure S6A). Transfection with WT or I96*V Bn-AFF (Supplementary Figures S6B, C) yielded very few fluorescent cells, suggesting those switches possessed significant RNase activity and therefore were mostly folded in the CP-frame. Consistent with this view, we observed many cells with dim fluorescence upon transfection with the I96*A mutant (Supplementary Figure S6D), and still more, brighter cells with the I96*G mutant (Supplementary Figure S6E). Cells expressing I96*G and WT constructs were comparably bright, implying that the I96*G mutation reduced RNase activity of Bn-AFF to baseline levels. Taken together, the results demonstrate that the Bn-AFF switch could be tuned in cells by rational mutagenesis of a buried residue in one of the folds.

**FIGURE 4:**
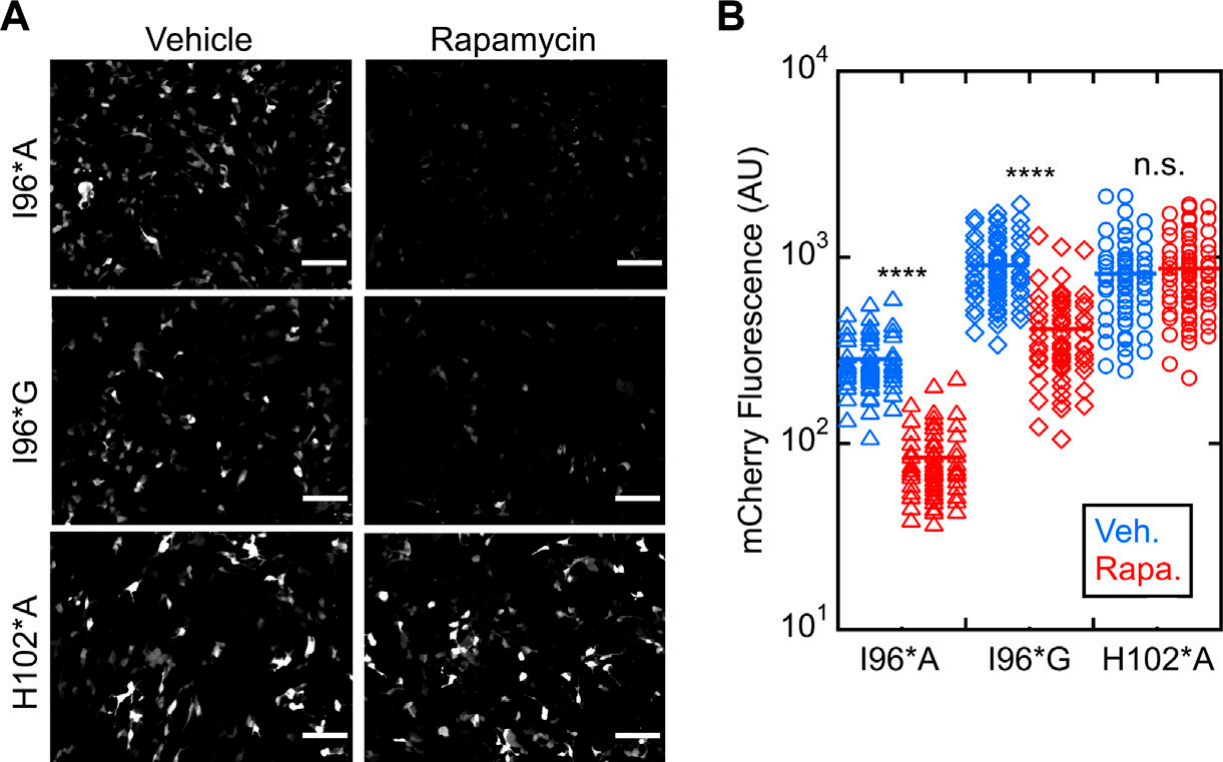
Bn-AFF degrades RNA in HEK293T cells upon binding rapamycin. **(A)** Raw images of cells co-transfected with one plasmid expressing mCherry-PEST reporter and another expressing Bn-AFF mutants I96*A, I96*G or H102*A control showed red fluorescence when treated with DMSO vehicle, signifying expression of mCherry mRNA. I96*A cells were less fluorescent than I96*G cells because I96*A had a lower ΔΔG_N/CP_ value than I96*G, increasing the baseline RNase activity of I96*A. After addition of 2.5 µM rapamycin, fluorescence decreased in I96*A and I96*G cells, indicating mCherry mRNA degradation. **(B)** Quantification revealed 3.4-fold and 2.2-fold decreases in fluorescence of I96*A and I96*G cells, respectively, in the presence of rapamycin compared to vehicle. No significant change was observed in the catalytically inactive control. Three images were used for quantification of mCherry fluorescence. The number of cells analyzed were 60 (I96*A, vehicle), 60 (I96*A, rapamycin), 58 (I96*G, vehicle), 62 (I96*G, rapamycin), 59 (H012*A, vehicle), and 65 (H102*A, rapamycin). Each dot represents a cell. A *t*-test with unequal variance was performed to test for significance: ****p < 10^−4^; ns, not statistically significant. Scalebar = 100 μm. The experiments shown are representative of four biological repeats. Mean ± s.d., for three biological repeats is reported in the text.

We next evaluated ligand-induced switching of I96*A and I96*G Bn-AFF mutants in HEK293T cells (WT and I96*V variants were dropped from further study due to their high background activities). Treating the I96*A and I96*G cells with rapamycin reduced the fluorescence significantly, suggesting activation of RNase activity (Figure 4A). Turn-on was quantified by selecting fluorescent cells and calculating the average intensity of each cell in the mCherry channel before and after rapamycin treatment (Figure 4B). I96*A and I96*G switches yielded (3.1 ± 0.3)-fold and (2.9 ± 0.7-fold) reductions in mCherry fluorescence, respectively (three biological repeats). No significant change in activity for the H102*A control was noted [(0.94 ± 0.03)-fold reduction; three biological repeats]. We therefore selected the I96*A and I96*G variants of Bn-AFF for *in vitro* characterization and cell viability studies.

### Validating Bn-AFF using purified protein

**FIGURE 5:**
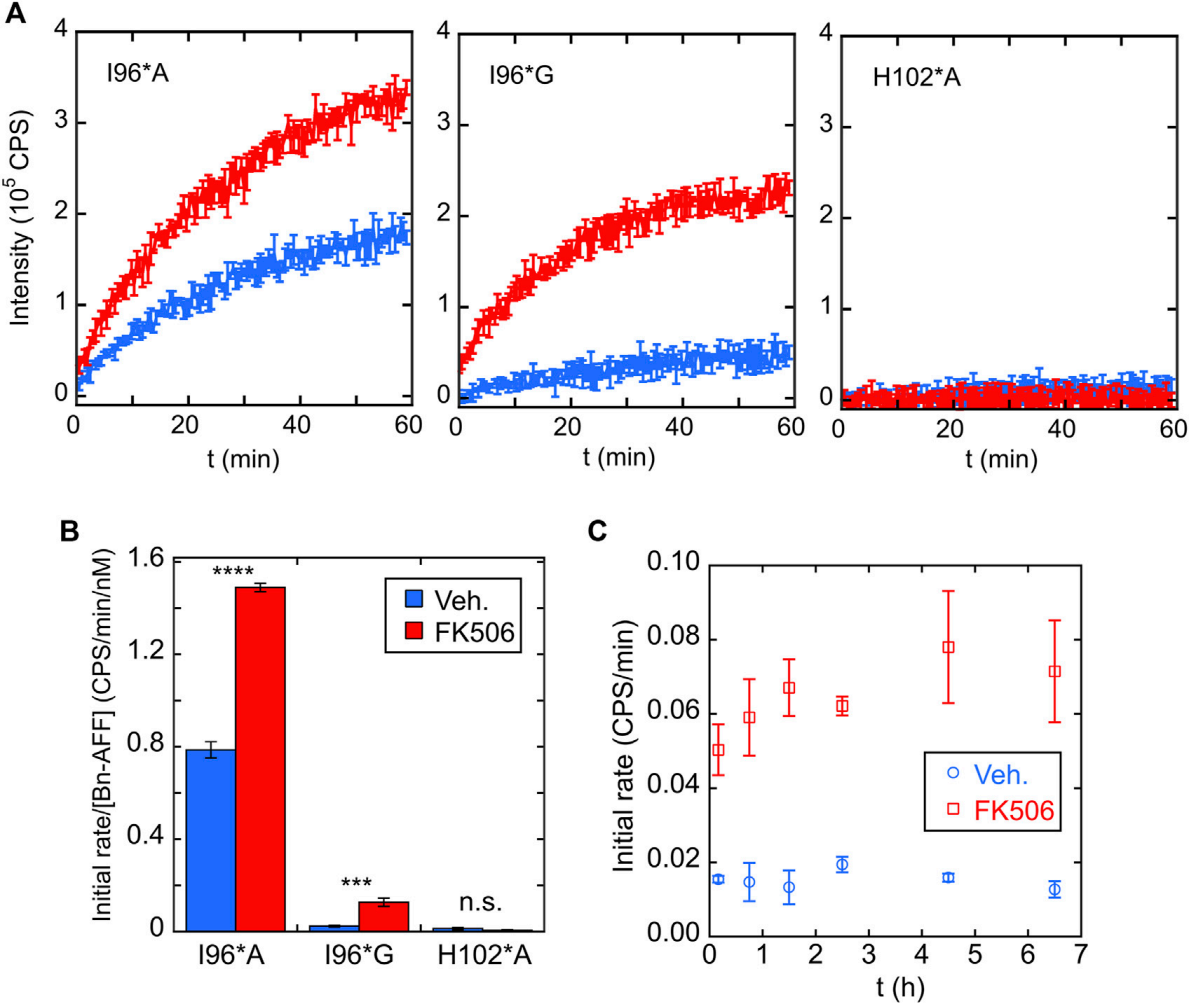
Bn-AFF shows ligand-dependent activation *in vitro.* **(A)** RNA hydrolysis in the absence of FK506 (blue curves) indicated that I96*G Bn-AFF had lower baseline enzymatic activity compared to I96*A, consistent with a higher ΔΔG_N/CP_ value for I96*G. Addition of FK506 (red curves) enhanced RNA hydrolysis by I96*A and I96*G. No significant RNA hydrolysis was observed for the control H102*A protein either in the absence or presence of FK506. **(B)** Quantification of FK506-dependent turn-on found 1.9 ± 0.1-fold and 5.3 ± 1.5-fold increases for I96*A and I96*G variants of Bn-AFF. Significance was evaluated using a *t*-test with unequal variance; ***p < 10^−3^; ****p < 10^−4^; n.s., not significant. **(C)** FK506 triggered the increase in enzymatic activity of I96*G variant mostly within the dead time of mixing, estimated to be ∼10 min. Data are represented as mean ± s.d., of three technical repeats.

We purified I96*A, I96*G, and H102*A Bn-AFF constructs from *Escherichia coli* and determined their enzymatic activities using the RNase Alert kit (Integrated DNA Technologies). As anticipated, in the absence of FK506, RNase activity of I96*A was greater than that of I96*G, with the latter value being close to background levels as judged by the H102*A control (Figure 5A). Addition of FK506 increased the RNA hydrolysis rates of I96*A and I96*G, demonstrating ligand-induced turn-on. No increase was observed for H102*A. Quantitating RNAse activity by initial velocity analysis revealed (1.9 ± 0.1)-fold and (5.3 ± 1.5)-fold turn-on values for I96*A and I96*G, respectively Figure 5B. These observations are consistent with the HEK293T cell data and support the switching mechanism in Figure 1D.

One aspect of AFF switching is that the fold shift can be slow (seconds to hours depending on the protein). This is because the switching rate is typically limited by unfolding of the N-fold (or CP-fold) which can be slow for stable proteins (Stratton and Loh, 2010; DeGrave et al., 2018; Bogetti et al., 2021). To determine the turn-on rate of Bn-AFF, we mixed the I96*G mutant with FK506, added RNA substrate at various times, and recorded the initial velocities. Interestingly, the fold shift reached completion within the dead time of the assay (∼5 min) Figure 5C. This fast rate was likely due to the modest thermodynamic stabilities of the N- and CP-frames, which lowered kinetic barriers for reaching equilibrium.

### Bn-AFF toxicity in yeast

Barnase has been previously reported to kill eukaryotic cells *via* caspase-mediated apoptosis (Edelweiss et al., 2008; Balandin et al., 2011). We detected no evidence that the Bn-AFF variants shown in Figure 4 were cytotoxic to HEK293T cells, either by MTT viability assays or by noting membrane blebbing or cell rounding/detachment (not shown). We speculated that lack of toxicity was due to a negative feedback loop in which Bn-AFF kept its expression at sublethal levels by degrading its own message, as we observed with mCherry mRNA (Figure 4). The previous studies cited above introduced the Bn protein directly into cells, perhaps at levels sufficient to cause death.

To evaluate the effect of Bn-AFF switching on eukaryotic cell viability, we used temperature to perturb the conformations of Bn-AFF expressed in *S. cerevisiae*. The haploid FKBP-deletion (*fpr1*Δ) strain was used to make the yeast resistant to rapamycin (Xu et al., 2010) and isolate potential cytotoxic effects to Bn-AFF activity. We first determined the melting temperatures (T_m_) of the CP-fold analogs containing the I96*A or I96*G mutations, in the presence of saturating FK506. These represent the active folds of the respective Bn-AFF mutants. At 37°C, the I96*G CP-fold analog (T_m_ = 23.2°C ± 0.8°C) was well beyond the unfolded baseline whereas the I96*A mutant (T_m_ = 30.5°C ± 0.2°C) was at the edge of the unfolding transition (Supplementary Figure S7). Reducing temperature to 30°C caused both I96G* and I96A* to substantially populate the folded state. We therefore reasoned that growing yeast at those two temperatures could provide a thermodynamic window for assessing rapamycin-induced switching and cell toxicity.

**FIGURE 6:**
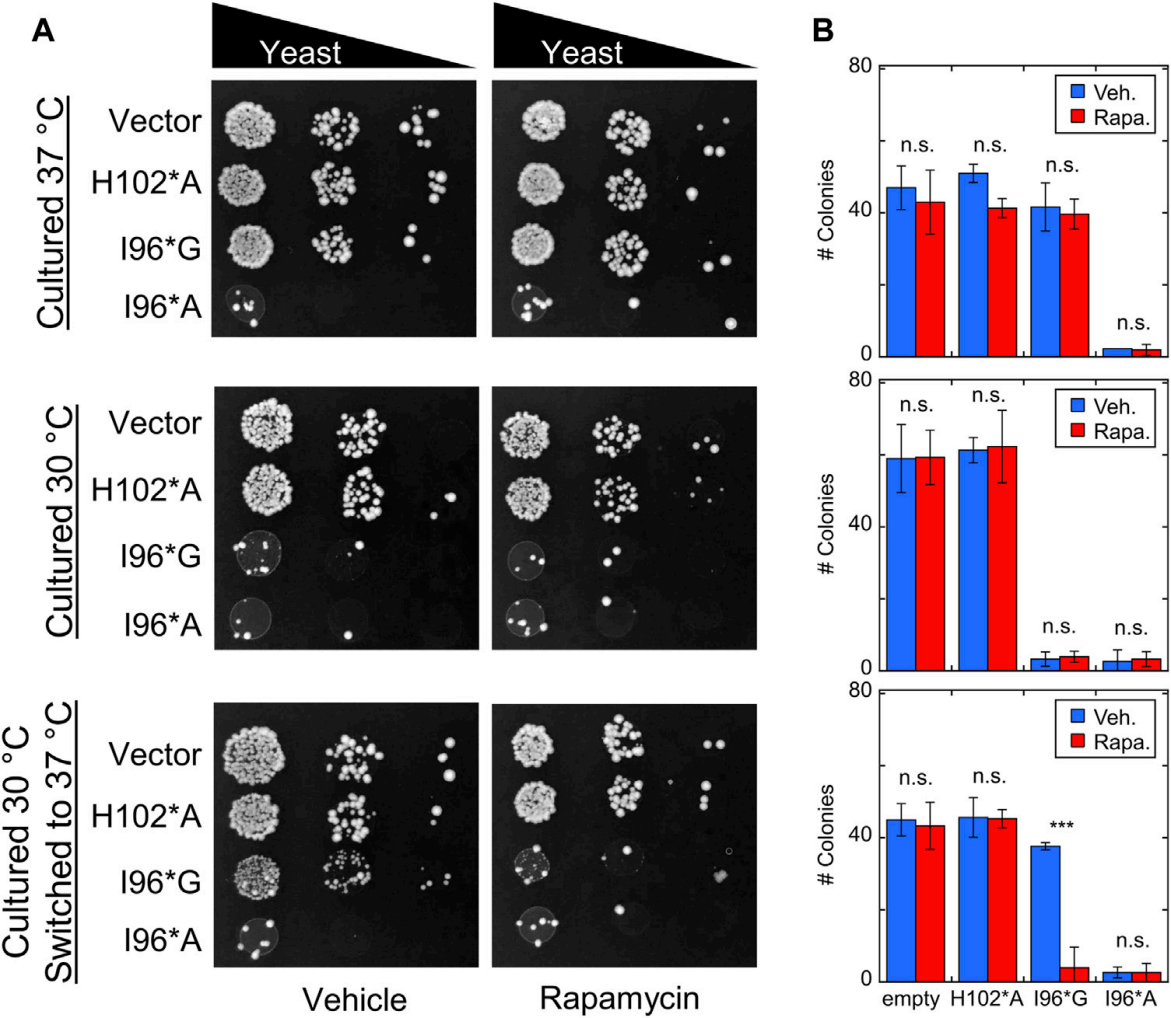
Bn-AFF demonstrates rapamycin-dependent toxicity in yeast. **(A)** Yeast cells were transformed with the indicated Bn-AFF constructs, spotted onto plates at 10, 100, and 1000-fold dilutions, and cultured at 30°C for 3 d (top row), 37°C for 3 d (middle row), or 30°C for 2 d then 37°C for 1 d (bottom row). I96*A was toxic at all three temperature conditions, whereas I96*G was toxic only at 30°C. I96*G exhibited rapamycin-dependent toxicity in cells that were shifted from 30°C to 37°C. **(B)** Rapamycin treatment resulted in significantly fewer colonies of I96*G cells grown at 30°C and shifted to 37°C, compared to vehicle alone. Rapamycin treatment had no effect on empty vector control cells or I96*A cells, likely because I96A* was already substantially active. The images are representative of three biological repeats with at least two technical repeats each. The numbers of colonies are mean ± s.d., of three technical repeats. Significance was evaluated using a *t*-test with unequal variance; ***p < 10^−3^; n.s., not significant.

Cells were transfected with Bn-AFF plasmids and allowed to grow for 3 days at 30°C or 37°C, with either rapamycin or DMSO vehicle added to the plates. Yeast expressing I96*A exhibited a slow growth phenotype at both temperatures (Figure 6), whereas I96*G cells grew at the same rate as the H102*A and empty vector controls. Rapamycin did not further increase toxicity in I96*A cells, suggesting that the CP-fold was already significantly populated at the two temperatures. The above results are in broad agreement with expectations from the thermal melts (Supplementary Figure S7).

I96*G cells grew normally at 37°C but slowly at 30°C, again consistent with its thermal stability (Supplementary Figure S6). Rapamycin did not reduce the growth rate of I96*G cells at 37°C, most likely because the CP-fold remained too unstable (at 14°C higher than its T_m_) relative to the N-fold to retain sufficient activity for cell toxicity. When I96*G cells cultured at 30°C were shifted to 37°C for 1 d, however, we observed evidence for rapamycin-induced switching. After shifting to 37°C, the surviving I96*G cells were able to grow approximately as rapidly as the H102*A and empty vector controls in the absence of rapamycin but failed to proliferate in the presence of the drug. This result indicates that rapamycin had indeed enhanced the activity of I96*G Bn-AFF, either at 30°C or after the shift to 37°C despite rapamycin having no effect on I96*G cells that were cultured continuously at 37°C. The latter case may be explained by the I96*G protein staying in the active CP-fold long enough after the temperature shift to exert cytotoxic effects. The former scenario may have arisen from the protein degrading more RNA in cells cultured with rapamycin compared to those treated with vehicle at 30°C, thus preventing or delaying their growth when shifted to the permissive temperature.

## Discussion

We developed a system that combines two forms of engineered allostery in proteins. A formalism was introduced for modeling how folding of two receptor domains, inserted into an AFF-modified POI, drives a ligand-dependent conformational change of the POI. Folding of one receptor domain stretches apart the N-fold of the POI (by MEF) and folding of a circularly permuted form of the same receptor domain concomitantly stabilizes the CP-fold of the POI (by LCE). These effects are additive and result in enhanced response of the switch toward the triggering ligand. The first example of this dual-action switch, Bn-AFF, showed ligand-dependent turn-on in human and yeast cells, and the mechanism was validated *in vitro*.

The above-described approach should be broadly applicable to the design of new switches, with the following primary considerations. For the AFF modification to be successful, the POI must be stable enough to tolerate insertion of the unstable receptor domain at a surface loop. It must also abide circular permutation. How to identify potential insertion and permutation positions in a POI has been described elsewhere (Ha and Loh, 2017; Dagliyan et al., 2018; Ha et al., 2019; Sekhon et al., 2022). To link switching to a change in biological activity, one of the duplicated sequences must contain a point mutation of a key functional residue or other modification. Obvious choices for point mutations are a catalytic residue to control enzymatic activity, or a binding residue to regulate protein-protein or protein-ligand interactions (Ha et al., 2013). We also fused a fluorescent protein to one of the duplicated segments of nanoluciferase to create a luminescent BRET biosensor that reported on DNA binding (Sekhon and Loh, 2022).

As to the receptor domains, the non-permuted version should have an N-to-C terminal distance of at least 15–20 Å when folded to disrupt the structure of the N-frame according to MEF. Both copies of the receptor domain must be unfolded when fused into their respective positions in the POI. On the MEF side, this condition is automatically satisfied if the POI is more stable than the non-permuted receptor, in which case the former forcibly unfolds the latter (Radley et al., 2003; Cutler et al., 2009). If not, a destabilizing mutation can be introduced into the receptor domain [e.g., the V24A mutation in FKBP which reduces stability by 3.2 kcal/mol (Fulton et al., 1999)]. For the LCE mechanism, the N-to-C terminal distance of the receptor domain should be short to ensure that it and the CP-fold are structurally compatible when they are both folded. Circularly permuting a protein guarantees a short N-to-C distance, and as an added benefit this process can be destabilizing enough to unfold the receptor domain [especially in concert with using short linkers (Butler et al., 2009; Sekhon and Loh, 2022)].

The simulations provide insight into the dual MEF-LCE mechanism and offer additional guidance for developing this class of switches. As one may intuit, ligand binding energy is the sole driving force behind MEF switching. An MEF switch can always be turned completely on given high enough ligand concentration and the assumption that the N-fold and the receptor domain cannot both be folded (this condition is established by the large N-to-C distance described above and is enforced in the simulations by a high MEF penalty in Supplementary Figure S1). LCE switching, by contrast, is driven by the LCE penalty. The LCE penalty is removed once cpFKBP is folded, and although adding more rapamycin will increase cpFKBP stability, no further stabilization of the CP-fold will be realized. Since the LCE penalty is theoretically proportional to the logarithm of the number of amino acids in a flexibly-jointed chain (Dagan et al., 2013), larger (and more disordered) receptor domains are expected to be more effective at inducing LCE switching. In this respect, intrinsically disordered proteins may be ideal for the LCE mechanism provided they adopt a single structure (or closely related ensemble of structures) upon binding a target ligand.

The simulations reveal that LCE excels at maximizing the number of molecules in the OFF state in the absence of ligand at the cost of incomplete conversion to the ON state at high [ligand]. MEF is the opposite: the ON state can be fully driven by binding, but this imposes practical limitations on the percentage of molecules that can be OFF without ligand. The model shows that the MEF-LCE switch combines the attributes of the individual mechanisms to enhance turn-on at all ligand concentrations.

The ability to control populations of OFF and ON states is crucial to tailoring a switch to the desired application. A high turn-on ratio is always desirable, but the absolute values of the ON fractions can also be an important consideration. For example, a 50-fold change from 1% ON to 50% ON in a fluorescent or luminescent biosensor makes for bright output and robust detection. However, 1% activity in the absence of ligand may be harmful to cells or animals if the switch is a cytotoxic enzyme such as barnase. In this case, changing the range from 0.1% ON to 5% ON reduces baseline activity by 10-fold with the same 50-fold turn-on ratio. The combined MEF-LCE mechanism, in concert with tuning ΔΔG_N/CP_ as described, affords latitude in adjusting these values.

The AFF mechanism developed here embodies the concept of ligand binding bringing about a distant conformational change, thereby introducing allosteric control into an otherwise unregulated protein. The phenomenon of binding-induced folding is widespread in natural and engineered proteins alike, suggesting our design may be generalizable. Indeed, previous examples of related AFF switches have used proteins that naturally bind ligands (Karchin et al., 2017; Gräwe and Merkx, 2022; John et al., 2022), proteins engineered to bind a specific ligand (Cutler et al., 2009), and even transcription factors to drive a fold shift (Ha et al., 2006; Sekhon and Loh, 2022). Moreover, although both binding domains recognized rapamycin in the present study, it is possible to use two proteins that target different ligands, allowing synergy to be programmed into the AFF design. Lastly, this mode of regulation is applicable to output proteins that perform a variety of functions; for example, staphylococcal nuclease (Karchin et al., 2017), ribose binding protein (Ha et al., 2012; Karchin et al., 2017), fluorescent proteins (John et al., 2022), and nanoluciferase (Sekhon and Loh, 2022). AFF in combination with MEF and LCE provides a pathway for modular design of protein switches.

## Materials and methods

### Gene construction and protein purification

For bacterial expression, C-terminal 6x-His tagged Bn-AFF constructs were cloned into the pETMT plasmid (Radley et al., 2003) downstream of the T7 promoter using NdeI/XhoI restriction sites. The pETMT vector contains barnase inhibitor barstar under a weak promoter to limit background activity of barnase. Proteins were expressed in *E. coli* BL21(DE3) and induced at OD_600_ 0.5–0.6 with 0.5 mM isopropyl β-D-thiogalactopyranoside for ≥16 h at 18°C. Cell pellets were resuspended in 20 mM Tris (pH 7.5), 0.5 M NaCl, 10 mM imidazole, and 6 M guanidine hydrochloride and lysed by sonication. Lysates were loaded onto a nickel-nitrilotriacetic column, and the column was washed with at 10 column volumes of lysis buffer before elution with lysis buffer supplemented with 0.3 M imidazole. The proteins were then extensively dialyzed against ddH_2_O and lyophilized.

For mammalian cell expression, Bn-AFF genes were cloned into the N1 vector (Clontech) downstream of the CMV promoter using AgeI/NotI sites. The proteins were tagged with a nuclear export signal to prevent barnase inhibition by DNA, and with yellow fluorescent protein to monitor transfection efficiency. For yeast expression, Bn-AFF genes were placed into the pBH787 vector (Gietz and Sugino, 1988) (gift from Brian K. Haarer) downstream of the TDH3 promoter using XbaI/SalI restriction sites. This vector contained the URA3 selection marker. All genes were fully sequenced and sequences are provided in Supplementary Note S1.

### Simulations of MEF and LCE models

Simulations were performed in GNU Octave using models in Supplementary Figures S1–S3. Full details are provided in Supplementary Note S2.

### HEK293T cell experiments

HEK293T cells were cultured at 37°C in DMEM containing 10% FBS. Cells were split into a 12-well plate 1 day before transfection at a confluency of 60%–80%. Plasmids containing the Bn-AFF genes and the mCherry-PEST reporter were mixed in equal amounts, and 1 μg of DNA was transfected using calcium phosphate method. Briefly, CaCl_2_ was added to the plasmid mix to 250 mM and equal amounts of 50 mM HEPES, 150 mM sodium phosphate, and 0.15 M NaCl (pH 7.0) were added dropwise while vortexing. The mixture was added directly to media after aging at room temperature for 20–30 min, and the media was replaced 15–20 h after transfection. After 36–48 h of expression, cells were washed with imaging media (DMEM, 50 mM HEPES pH 7.0, no phenol red), and 2.5 μM rapamycin or 0.025% DMSO vehicle was added to the imaging media.

After 12–15 h of rapamycin exposure, cells were mounted on imaging media and imaged with a Zeiss Axioimager Z1 upright microscope (10X/0.25 A-Plan Objective) equipped with a mercury lamp. mCherry fluorescence images were obtained using a 560 ± 40 nm excitation filter and a 630 ± 75 nm emission filter. YFP fluorescence was used to randomly identify regions with >50 cells and at least 3 fields were imaged. Cell images were quantified using Fiji (Schindelin et al., 2012). Background was subtracted using the built-in plugin using 50 pixels as the sliding ball radius. No further processing was applied, and the intensity of each cell was measured by manually drawing a region of interest around each cell and determining the average intensity using the Measure Particles plugin.

### Stability determination

For urea denaturation experiments, lyophilized proteins were dissolved in 50 mM HEPES (pH 7.0), 100 mM NaCl, 6 M Urea, and 0.01% Tween-20. The solution was then rapidly diluted (∼200-fold, to a final protein concentration of 0.5–1.0 µM) into the same buffer and to the buffer that lacked urea. FK506 (20 µM) or the equivalent volume of DMSO was added to both solutions, and the solutions were mixed in various ratios using a Hamilton Microlab 540B dispenser. Urea concentration was measured by refractive index. After mixing, the samples were equilibrated at 4°C for 12–15 h and their tryptophan fluorescence spectra was recorded on a Horiba Fluoromax-4 fluorometer (280 nm excitation, 320–450 nm emission, 4°C). The maximum emission wavelength was determined by fitting the spectra to an exponentially modified gaussian function using Igor Pro (Wavemetrics Inc.). The maximum emission as a function of [urea] was then fit to the 2-state linear extrapolation equation (Santoro and Bolen, 1988) to obtain ΔG_unfold_, C_m_, and *m*-value using Kaleidagraph (Synergy Software).

Thermal denaturation experiments were performed by recording fluorescence spectra every 2°C from 10°C–50°C with ∼30 s equilibration between temperatures, using the same instrument settings described above. Solution conditions were 0.5 µM protein, 20 mM sodium phosphate (pH 7.0), 100 mM NaCl, and 20 µM FK506. T_m_ values were determined by plotting the derivative of the maximum emission wavelength as a function of temperature and fitting to a gaussian function using Kaleidagraph.

### RNase activity assays

Lyophilized proteins were dissolved in 6 M GdnHCl and rapidly diluted to 20 nM into 50 mM HEPES (pH 7.0), 100 mM NaCl, 0.005% Tween-20. After equilibrating at room temperature for at least 20 m, 50 μM FK506 or an equivalent amount of DMSO vehicle was added and the solutions were kept overnight at room temperature. The solutions were then diluted to 0.1–1.0 nM and 20 nM of RNaseAlert substrate (IDT technologies) was added. Fluorescence changes were recorded using a SpectraMax i3x plate reader (Molecular Devices) with the following conditions: 96-well fully opaque plate, 150 μL per well, 1 mm read height, 485 nm excitation, 535 nm emission, and 30 s interval between measurements). Background signal was subtracted using a well containing only substrate in buffer. The first 7–10 m of the reactions were fit to a linear equation to determine initial rate.

### Yeast experiments

The rapamycin-resistant haploid yeast strain (*MAT*a *ura3∆0 leu2∆0 his3∆1 fpr1∆::G418* ^*R*^) was transformed with the plasmid containing Bn-AFF using the lithium acetate/PEG method (Gietz and Schiestl, 2007) with sheared salmon sperm DNA as the carrier. Transformed cells were directly spotted onto plates containing 7 g/L yeast nitrogen base without amino acids, 0.25% casamino acids, 0.05 mg/mL tryptophan, 0.0025 mg/ml adenine, and either 1 μM rapamycin or 0.005% DMSO vehicle. The plates were supplemented with additional rapamycin or DMSO by spreading the solution once dry and drying them further for 3 h at 37°C. The cells were diluted by 10, 100, and 1000-fold, and 5 μL of each dilution was spotted onto the plates. The 100-fold dilutions were chosen for quantification by manual counting. Empty vector (plasmid containing no barnase gene) and no vector (only salmon sperm DNA) served as controls in all experiments.

### Reproducibility, statistics, and data availability

All experiments described in this study were performed a minimum of three times (technical repeats). The mammalian cell and yeast experiments consisted of three biological repeats with new cells freshly split and transformed on different days. For activity assays, the I96*G mutant was purified two separate times. The turn-on ratios (determined from multiple technical repeats) were identical within error. Data are plotted as mean ± s.d. The statistical significance, where stated, was determined by a *t*-test with unequal variance using Kaleidagraph. All data and code generated in this paper is available on the Mendeley database (DOI 10.17632/j2rm8p4jr5.1). The plasmids will be made available upon request.

## Data Availability

The original contributions presented in the study are included in the article/Supplementary Material, further inquiries can be directed to the corresponding author. https://data.mendeley.com/datasets/j2rm8p4jr5. DOI:10.17632/j2rm8p4jr5.1.

## References

[B1] Balandin T. G. Edelweiss E. Andronova N. V. Treshalina E. M. Sapozhnikov A. M. Deyev S. M. (2011). Antitumor activity and toxicity of anti-HER2 immunoRNase scFv 4D5-dibarnase in mice bearing human breast cancer xenografts. Invest. New Drugs 29, 22–32. 10.1007/s10637-009-9329-2 19789841

[B2] Bogetti A. T. Presti M. F. Loh S. N. Chong L. T. (2021). The next frontier for designing switchable proteins: Rational enhancement of kinetics. J. Phys. Chem. B 125, 9069–9077. 10.1021/acs.jpcb.1c04082 34324338 PMC8826494

[B3] Butler J. S. Mitrea D. M. Mitrousis G. Cingolani G. Loh S. N. (2009). Structural and thermodynamic analysis of a conformationally strained circular permutant of barnase. Biochemistry 48, 3497–3507. 10.1021/bi900039e 19260676 PMC2756614

[B5] Cutler T. A. Loh S. N. (2007). Thermodynamic analysis of an antagonistic folding-unfolding equilibrium between two protein domains. J. Mol. Biol. 371, 308–316. 10.1016/j.jmb.2007.05.077 17572441 PMC2041865

[B6] Cutler T. A. Mills B. M. Lubin D. J. Chong L. T. Loh S. N. (2009). Effect of interdomain linker length on an antagonistic folding–unfolding equilibrium between two protein domains. J. Mol. Biol. 386, 854–868. 10.1016/j.jmb.2008.10.090 19038264 PMC2756608

[B7] Dagan S. Hagai T. Gavrilov Y. Kapon R. Levy Y. Reich Z. (2013). Stabilization of a protein conferred by an increase in folded state entropy. Proc. Natl. Acad. Sci. 110, 10628–10633. 10.1073/pnas.1302284110 23754389 PMC3696814

[B8] Dagliyan O. Krokhotin A. Ozkan-Dagliyan I. Deiters A. Der C. J. Hahn K. M. (2018). Computational design of chemogenetic and optogenetic split proteins. Nat. Commun. 9, 4042. 10.1038/s41467-018-06531-4 30279442 PMC6168510

[B9] Dagliyan O. Tarnawski M. Chu P.-H. Shirvanyants D. Schlichting I. Dokholyan N. V. (2016). Engineering extrinsic disorder to control protein activity in living cells. Science 354, 1441–1444. 10.1126/science.aah3404 27980211 PMC5362825

[B10] DeGrave A. J. Ha J.-H. Loh S. N. Chong L. T. (2018). Large enhancement of response times of a protein conformational switch by computational design. Nat. Commun. 9, 1013. 10.1038/s41467-018-03228-6 29523842 PMC5844902

[B11] Diaz J. E. Morgan C. W. Minogue C. E. Hebert A. S. Coon J. J. Wells J. A. (2017). A split-abl kinase for direct activation in cells. Cell Chem. Biol. 24, 1250–1258.e4. 10.1016/j.chembiol.2017.08.007 28919041 PMC5650542

[B12] Do K. Boxer S. G. (2013). GFP variants with alternative β-strands and their application as light-driven protease sensors: A tale of two tails. J. Am. Chem. Soc. 135, 10226–10229. 10.1021/ja4037274 23819615 PMC3756597

[B13] Edelweiss E. Balandin T. G. Ivanova J. L. Lutsenko G. V. Leonova O. G. Popenko V. I. (2008). Barnase as a new therapeutic agent triggering apoptosis in human cancer cells. PLOS ONE 3, e2434. 10.1371/journal.pone.0002434 18560598 PMC2413406

[B14] Fulton K. F. Main E. R. Daggett V. Jackson S. E. (1999). Mapping the interactions present in the transition state for unfolding/folding of FKBP12. J. Mol. Biol. 291, 445–461. 10.1006/jmbi.1999.2942 10438631

[B15] Gietz R. D. Schiestl R. H. (2007). High-efficiency yeast transformation using the LiAc/SS carrier DNA/PEG method. Nat. Protoc. 2, 31–34. 10.1038/nprot.2007.13 17401334

[B16] Gietz R. D. Sugino A. (1988). New yeast-Escherichia coli shuttle vectors constructed with *in vitro* mutagenized yeast genes lacking six-base pair restriction sites. Gene 74, 527–534. 10.1016/0378-1119(88)90185-0 3073106

[B17] Gräwe A. Merkx M. (2022). Bioluminescence goes dark: Boosting the performance of bioluminescent sensor proteins using complementation inhibitors. ACS Sens. 7, 3800–3808. 10.1021/acssensors.2c01726 36450135 PMC9791688

[B18] Guo Z. Parakra R. D. Xiong Y. Johnston W. A. Walden P. Edwardraja S. (2022). Engineering and exploiting synthetic allostery of NanoLuc luciferase. Nat. Commun. 13, 789. 10.1038/s41467-022-28425-2 35145068 PMC8831504

[B19] Ha J.-H. Butler J. S. Mitrea D. M. Loh S. N. (2006). Modular enzyme design: Regulation by mutually exclusive protein folding. J. Mol. Biol. 357, 1058–1062. 10.1016/j.jmb.2006.01.073 16483603 PMC3145369

[B20] Ha J.-H. Karchin J. M. Walker-Kopp N. Castañeda C. A. Loh S. N. (2015). Engineered domain swapping as an on/off switch for protein function. Chem. Biol. 22, 1384–1393. 10.1016/j.chembiol.2015.09.007 26496687 PMC4621486

[B21] Ha J.-H. Karchin J. M. Walker-Kopp N. Huang L.-S. Berry E. A. Loh S. N. (2012). Engineering domain swapped binding interfaces by mutually exclusive folding. J. Mol. Biol. 416, 495–502. 10.1016/j.jmb.2011.12.050 22245575 PMC3288482

[B22] Ha J.-H. Loh S. N. (2017). Construction of allosteric protein switches by alternate frame folding and intermolecular fragment exchange. Methods Mol. Biol. Clifton N. J. 1596, 27–41. 10.1007/978-1-4939-6940-1_2 PMC5538016 28293878

[B23] Ha J.-H. Presti M. F. Loh S. N. (2019). A single protein disruption site results in efficient reassembly by multiple engineering methods. Biophys. J. 117, 56–65. 10.1016/j.bpj.2019.06.002 31221439 PMC6626842

[B24] Ha J.-H. Shinsky S. A. Loh S. N. (2013). Stepwise conversion of a binding protein to a fluorescent switch: Application to thermoanaerobacter tengcongensis ribose binding protein. Biochemistry 52, 600–612. 10.1021/bi301105u 23302025 PMC5966831

[B25] John A. M. Sekhon H. Ha J.-H. Loh S. N. (2022). Engineering a fluorescent protein color switch using entropy-driven β-strand exchange. ACS Sens. 7, 263–271. 10.1021/acssensors.1c02239 35006676 PMC10351476

[B26] Karchin J. M. Ha J.-H. Namitz K. E. Cosgrove M. S. Loh S. N. (2017). Small molecule-induced domain swapping as a mechanism for controlling protein function and assembly. Sci. Rep. 7, 44388. 10.1038/srep44388 28287617 PMC5347425

[B27] Minard P. Scalley-Kim M. Watters A. Baker D. (2001). A "loop entropy reduction" phage-display selection for folded amino acid sequences. Protein Sci. Publ. Protein Soc. 10, 129–134. 10.1110/ps.32401 PMC2249851 11266601

[B28] Mitrea D. M. Parsons L. S. Loh S. N. (2010). Engineering an artificial zymogen by alternate frame protein folding. Proc. Natl. Acad. Sci. 107, 2824–2829. 10.1073/pnas.0907668107 20133757 PMC2840272

[B29] Ni Y. Arts R. Merkx M. (2019). Ratiometric bioluminescent sensor proteins based on intramolecular split luciferase complementation. ACS Sens. 4, 20–25. 10.1021/acssensors.8b01381 30525479 PMC6350203

[B30] Radley T. L. Markowska A. I. Bettinger B. T. Ha J.-H. Loh S. N. (2003). Allosteric switching by mutually exclusive folding of protein domains. J. Mol. Biol. 332, 529–536. 10.1016/s0022-2836(03)00925-2 12963365 PMC3145375

[B31] Santoro M. M. Bolen D. W. (1988). Unfolding free energy changes determined by the linear extrapolation method. 1. Unfolding of phenylmethanesulfonyl alpha-chymotrypsin using different denaturants. Biochemistry 27, 8063–8068. 10.1021/bi00421a014 3233195

[B32] Scalley-Kim M. Minard P. Baker D. (2003). Low free energy cost of very long loop insertions in proteins. Protein Sci. 12, 197–206. 10.1110/ps.0232003 12538883 PMC2312413

[B33] Schindelin J. Arganda-Carreras I. Frise E. Kaynig V. Longair M. Pietzsch T. (2012). Fiji: An open-source platform for biological-image analysis. Nat. Methods 9, 676–682. 10.1038/nmeth.2019 22743772 PMC3855844

[B34] Sekhon H. Ha J.-H. Loh S. N. (2022). “Chapter One - engineering protein and DNA tools for creating DNA-dependent protein switches,” in Methods in enzymology, integrated methods in protein biochemistry: Part A. Editor Shukla A. K. (Academic Press), 1–32. 10.1016/bs.mie.2022.07.002 PMC10314797 36220266

[B35] Sekhon H. Loh S. N. (2022). Engineering protein activity into off-the-shelf DNA devices. Cell Rep. Methods 2, 100202. 10.1016/j.crmeth.2022.100202 35497497 PMC9046454

[B36] Stratton M. M. Loh S. N. (2011). Converting a protein into a switch for biosensing and functional regulation. Protein Sci. Publ. Protein Soc. 20, 19–29. 10.1002/pro.541 PMC3047058 21064163

[B37] Stratton M. M. Loh S. N. (2010). On the mechanism of protein fold-switching by a molecular sensor. Proteins 78, 3260–3269. 10.1002/prot.22833 20806404 PMC3145374

[B38] Stratton M. M. Mitrea D. M. Loh S. N. (2008). A Ca2+-sensing molecular switch based on alternate frame protein folding. ACS Chem. Biol. 3, 723–732. 10.1021/cb800177f 18947182 PMC2769504

[B39] Woloschuk R. M. Reed P. M. M. Jaikaran A. S. I. Demmans K. Z. Youn J. Kanelis V. (2021). Structure-based design of a photoswitchable affibody scaffold. Protein Sci. 30, 2359–2372. 10.1002/pro.4196 34590762 PMC8605370

[B40] Wood R. J. Ormsby A. R. Radwan M. Cox D. Sharma A. Vöpel T. (2018). A biosensor-based framework to measure latent proteostasis capacity. Nat. Commun. 9, 287. 10.1038/s41467-017-02562-5 29348634 PMC5773518

[B41] Xu T. Johnson C. A. Gestwicki J. E. Kumar A. (2010). Conditionally controlling nuclear trafficking in yeast by chemical-induced protein dimerization. Nat. Protoc. 5, 1831–1843. 10.1038/nprot.2010.141 21030958 PMC4976631

[B42] Zetsche B. Volz S. E. Zhang F. (2015). A split-Cas9 architecture for inducible genome editing and transcription modulation. Nat. Biotechnol. 33, 139–142. 10.1038/nbt.3149 25643054 PMC4503468

