# Supplementary material for: Enhancing response of a protein conformational switch by using two disordered ligand binding domains
Source: Front Mol Biosci. 2023 Mar 2;10:1114756. doi: 10.3389/fmolb.2023.1114756 (PMC10018487; doi:10.3389/fmolb.2023.1114756)
Supplement: Supplementary file 1 [file DataSheet1.DOCX]

Supplementary Material

**Enhancing Response of a Protein Conformational Switch by Using Two Disordered Ligand Binding Domains**

Harsimranjit Sekhon, Jeung-Hoi Ha, Stewart N. Loh*

* Address correspondence to S.N.L at lohs@upstate.edu

**Supplementary Note 1.** **Amino acid sequences of the Bn-AFF constructs**

**Supplementary Note 2. Simulation of MEF, LCE, and combined MEF-LCE switching**

**Supplementary Figure S1. MEF-only switching simulation, supporting Fig. 2**

**Supplementary Figure S2. LCE-only switching simulation, supporting Fig. 2**

**Supplementary Figure S3. Simulation of combined MEF-LCE switching, supporting Fig. 2**

**Supplementary Figure S4. Fractional changes of significantly populated states in the simulations, plotted as functions of [rapamycin] and ΔΔG_N/CP_, supporting Fig. 2**

**Supplementary Figure S5. Raw data from urea denaturation experiments, supporting Fig. 3.**

**Supplementary Figure S6. Thermodynamic tuning of Bn-AFF in HEK293T cells, supporting Fig. 4**

**Supplementary Figure S7. Thermal denaturation curves for the CP-fold analogs of I96*G and I96*A mutants, supporting Fig. 6**

**Supplementary Table 1. Thermodynamic parameters from urea denaturation experiments, supporting Fig. 3**

**Supplementary Note 1: Amino acid sequences of Bn-AFF constructs**

Sequences are color-coded according to Fig. 1**:**

**Bn(67-110)—cpFKBP—Bn(1-66)—FKBP—Bn(67-110)**

**BnAFF(H102*A)** Tuning Mutation; Activity Mutation (CP); Activity Mutation (N Frame) MSGRTWREADINYTSGFRNSDRILYSSDWLIYKTTDAYQTFTKIRGARGGSGKKFDSSRDRNKPFKFMLGKQEVIRGWEEGVAQMSVGQRAKLTISPDYAYGATGHPGIIPPHATLVFDVELLKLEGGAASGGAAGGSSGAASSGAGAAGGSGAGGGVQVETISPGDGRTFPKRGQTAVAHYTGMLEDIGSGGSGKSAGGMAQVINTFDGVADYLQTYHKLPDNYITKSEAQALGWVASKGNLADVAPGKSIGGDIFSNREGKLPGKGVQVETISPGDGRTFPKRGQTAVAHYTGMLEDGKKFDSSRDRNKPFKFMLGKQEVIRGWEEGVAQMSVGQRAKLTISPDYAYGATGHPGIIPPHATLVFDVELLKLESGRTWREADINYTSGFRNSDRILYSSDWLIYKTTDAYQTFTKIR

**BnAFF(I96*A)** Tuning Mutation; Activity Mutation (CP); Activity Mutation (N Frame) MSGRTWREADINYTSGFRNSDRILYSSDWLAYKTTDHYQTFTKIRGARGGSGKKFDSSRDRNKPFKFMLGKQEVIRGWEEGVAQMSVGQRAKLTISPDYAYGATGHPGIIPPHATLVFDVELLKLEGGAASGGAAGGSSGAASSGAGAAGGSGAGGGVQVETISPGDGRTFPKRGQTAVAHYTGMLEDIGSGGSGKSAGGMAQVINTFDGVADYLQTYHKLPDNYITKSEAQALGWVASKGNLADVAPGKSIGGDIFSNREGKLPGKGVQVETISPGDGRTFPKRGQTAVAHYTGMLEDGKKFDSSRDRNKPFKFMLGKQEVIRGWEEGVAQMSVGQRAKLTISPDYAYGATGHPGIIPPHATLVFDVELLKLESGRTWREADINYTSGFRNSDRILYSSDWLIYKTTDAYQTFTKIR

**BnAFF(I96*G)** Tuning Mutation; Activity Mutation (CP); Activity Mutation (N Frame) MSGRTWREADINYTSGFRNSDRILYSSDWLGYKTTDHYQTFTKIRGARGGSGKKFDSSRDRNKPFKFMLGKQEVIRGWEEGVAQMSVGQRAKLTISPDYAYGATGHPGIIPPHATLVFDVELLKLEGGAASGGAAGGSSGAASSGAGAAGGSGAGGGVQVETISPGDGRTFPKRGQTAVAHYTGMLEDIGSGGSGKSAGGMAQVINTFDGVADYLQTYHKLPDNYITKSEAQALGWVASKGNLADVAPGKSIGGDIFSNREGKLPGKGVQVETISPGDGRTFPKRGQTAVAHYTGMLEDGKKFDSSRDRNKPFKFMLGKQEVIRGWEEGVAQMSVGQRAKLTISPDYAYGATGHPGIIPPHATLVFDVELLKLESGRTWREADINYTSGFRNSDRILYSSDWLIYKTTDAYQTFTKIR

For bacterial and yeast vectors, the constructs were tagged with a 6xHis at the C-terminus, connected by an HRV3C protease cleavage site:

**BnAFF**-LEAGGLEVLFQGPAAAHHHHHH

For the mammalian expression vectors below, a nuclear export signal along with a YFP were tagged at the N-terminus:

MNLVDLQKKLEELELDEQQGPVATMVSKGEELFTGVVPILVELDGDVNGHKFSVSGEGEGDATYGKLTLKFICTTGKLPVPWPTLVTTFGYGLQCFARYPDHMKQHDFFKSAMPEGYVQERTIFFKDDGNYKTRAEVKFEGDTLVNRIELKGIDFKEDGNILGHKLEYNYNSHNVYIMADKQKNGIKVNFKIRHNIEDGSVQLADHYQQNTPIGDGPVLLPDNHYLSYQSALSKDPNEKRDHMVLLEFVTAAGITLGMDELYKSAGG-**BnAFF**

mCherry-PEST: MVSKGEEDNMAIIKEFMRFKVHMEGSVNGHEFEIEGEGEGRPYEGTQTAKLKVTKGGPLPFAWDILSPQFMYGSKAYVKHPADIPDYLKLSFPEGFKWERVMNFEDGGVVTVTQDSSLQDGEFIYKVKLRGTNFPSDGPVMQKKTMGWEASSERMYPEDGALKGEIKQRLKLKDGGHYDAEVKTTYKAKKPVQLPGAYNVNIKLDITSHNEDYTIVEQYERAEGRHSTGGMDELYKNSHGFPPEVEEQAAGTLPMSCAQESGMDRHPAACASARINV

**Supplementary Note 2: Simulation of MEF, LCE, and combined MEF-LCE switching**

***Generation of equilibrium folding models and nomenclature.*** Equilibrium models were formulated by first enumerating all possible states of the switches. States of the full Bn-AFF switch are represented by a three-position code (cpFKBP-Bn-FKBP) indicating the conformational state of each of the three domains. The first position can be N or U, corresponding to folded and unfolded states of the cpFKBP domain. The central position can be N, CP, or U, corresponding to the N-fold, CP-fold, and unfolded forms of the Bn domain. The third position can be N or U, corresponding to folded and unfolded states of the FKBP domain. The central position dictates whether the switch is ON or OFF and is highlighted by a box. For example, N-CP-U refers to the state in which cpFKBP is folded, Bn is in the CP-fold (ON state), and FKBP is unfolded.

Reactions that involve folding/unfolding of a single domain within the fusion protein were then assigned a free energy of unfolding (ΔG_unfold_). The ΔG_unfold_ term represents the intrinsic stability of that domain, *i.e.*, the free energy of unfolding in the absence of the other domain to which its fused. In some reactions, unfolding of one domain is made more favorable when the other domain is folded (or unfolded) and this is the heart of the MEF and LCE phenomena. These effects are modeled by adding an additional free energy term (the MEF or LCE penalties) to those reactions.

In all simulations, FKBP and cpFKBP were assigned identical ΔG_unfold_ values of -2 kcal/mol (and identical K_d_ values of 1 nM). The remaining ΔG_unfold_ terms were those of the N-fold and CP-fold. Most of the simulations entailed initially setting these to the same value (5 kcal/mol), and then changing ΔG_unfold_ of the CP-fold to 4, 3, and 2 kcal/mol to simulate the effect of tuning mutations. ΔΔG_N/CP_ is defined as ΔG_unfold_ (N-fold) minus ΔG_unfold_ (CP-fold), and hence are 0, 1, 2, and 3 kcal/mol, respectively.

***Simulation methods.*** Populations of all species were calculated in GNU Octave (www.octave.org) by creating an *m* x *n* matrix (columns x rows) where *m* is the number of total states + 1, and *n* is the total number of equilibrium constants + 1. The first row was set to all 1 to indicate that the populations of all states sum to unity. The remaining rows were filled according to the equilibrium constants derived from the ΔG_unfold_ values described above. As an example, the below matrix highlighted in yellow was used for the MEF model (Fig. S1), where K_AC_ = exp(ΔG_AC_/RT), etc., and T = 283 K:

|  | A | B | C | G | H | Total |  | Interpretation |
| --- | --- | --- | --- | --- | --- | --- | --- | --- |
| 0 | 1 | 1 | 1 | 1 | 1 | 1 |  | [A]+[B]+[C]+[G]+[H]=1 |
| 1 | -K_AC_ | 0 | 1 | 0 | 0 | 0 |  | [C]-K_1_*[A]=0 |
| 2 | 0 | 0 | -K_CH_ | 0 | 1 | 0 |  | [H]-K_2_*[C]=0 |
| 0 | 0 | 0 | 0 | -K_GH_ | 1 | 0 |  | [H]-K_3_*[G]=0 |
| 4 | -K_AG_ | 0 | 0 | 1 | 0 | 0 |  | [G]-K_4_*[A]=0 |
| 5 | -K_AB_ | 1 | 0 | 0 | 0 | 0 |  | [B]-K_5_*[A]=0 |

To simulate rapamycin binding to the folded states of FKBP and cpFKBP, their intrinsic unfolding free energies were modified by adding the term RT·ln(1+K_d_^-1^[rapamycin]). The operation ‘rref’ was then performed on the matrices to generate the populations of each species. The fraction of molecules in the ON state was calculated by summing all fractions in which the CP-fold was folded, whether bound by rapamycin or not.


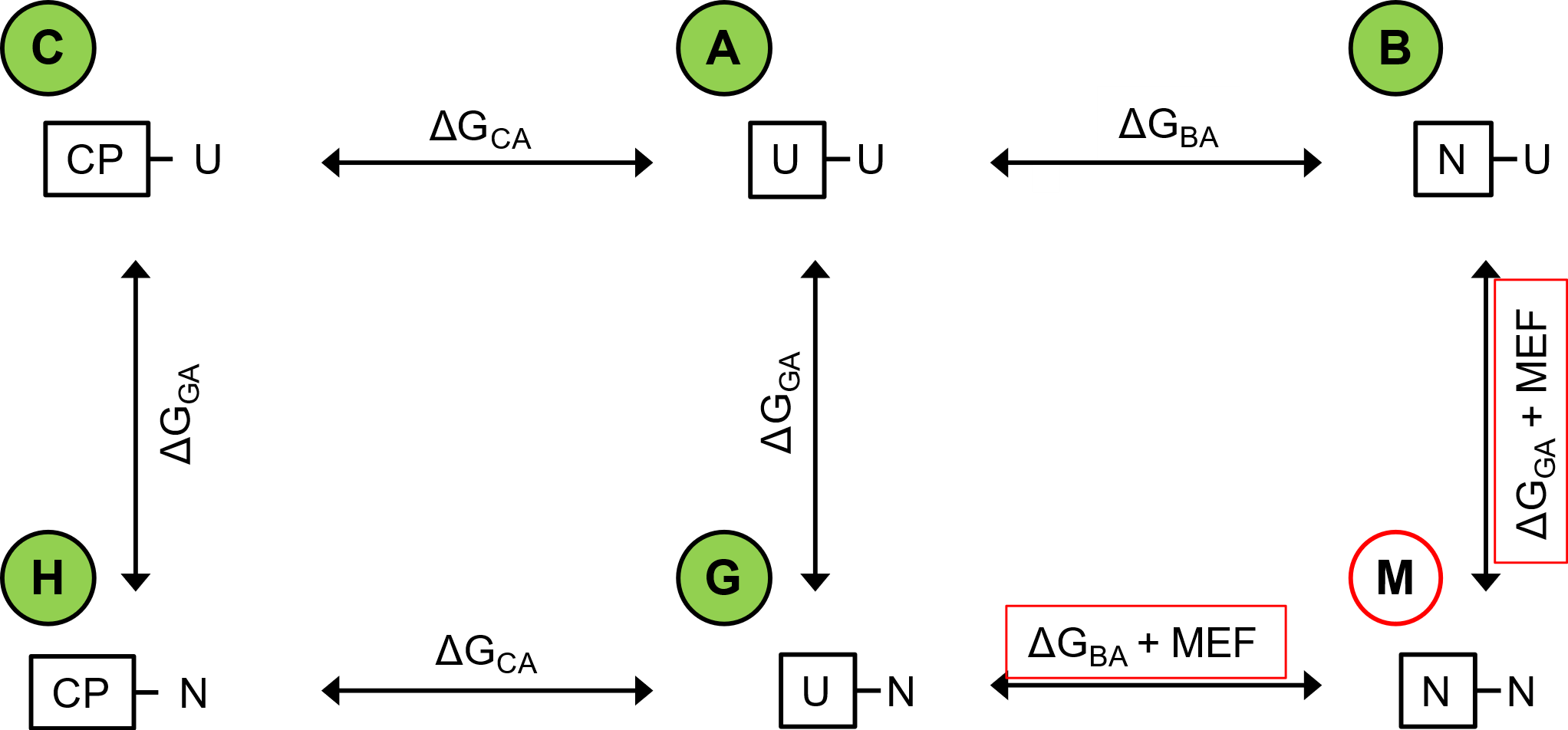


**Supplemental Figure S1.** **MEF-only switching simulation, supporting Fig. 2.** The MEF-only switch (Fig. 1B) consists of FKBP inserted into the N-fold of Bn-AFF, yielding 6 states. Since cpFKBP is not present in this construct, the states are represented by a two-letter code corresponding to Bn-FKBP. The MEF penalty is imposed on any folding reaction (red boxes) that produces the N-N state (**M**). We assumed the MEF penalty to be so large so that **M** is not populated at any condition. Because FKBP is unfolded in the absence of rapamycin, the molecules initially populate the upper row according to the relative stabilities of the N-fold and CP-fold (ΔΔG_N/CP_). As the N-fold becomes more stable (ΔΔG_N/CP_ increases from zero to 3 kcal/mol in Fig. 2), the molecules flow from equal populations **C** (ON) and **B** (OFF) to almost entirely **B** (**M** is also stabilized, but it is disallowed). When rapamycin is added, it stabilizes any state that contains folded FKBP (lower row). The molecules flow almost exclusively into **H**, the most stable state in the lower row, and the switch turns ON. Changes in the distributions of populations as functions of [rapamycin] and ΔΔG_N/CP_ are shown in Fig. S4A**.** The following ΔG_unfold_ values (in units of kcal/mol) were used to generate the data in in Fig. 2 and Fig. S4A: ΔG_BA_ = 5; ΔG_GA_ = -2+ RT·ln(1+K_d_^-1^[rapamycin]); ΔG_CA_ was set to 5, 4, 3, and 2 to make ΔΔG_N/CP_ = 0, 1, 2, and 3.

**
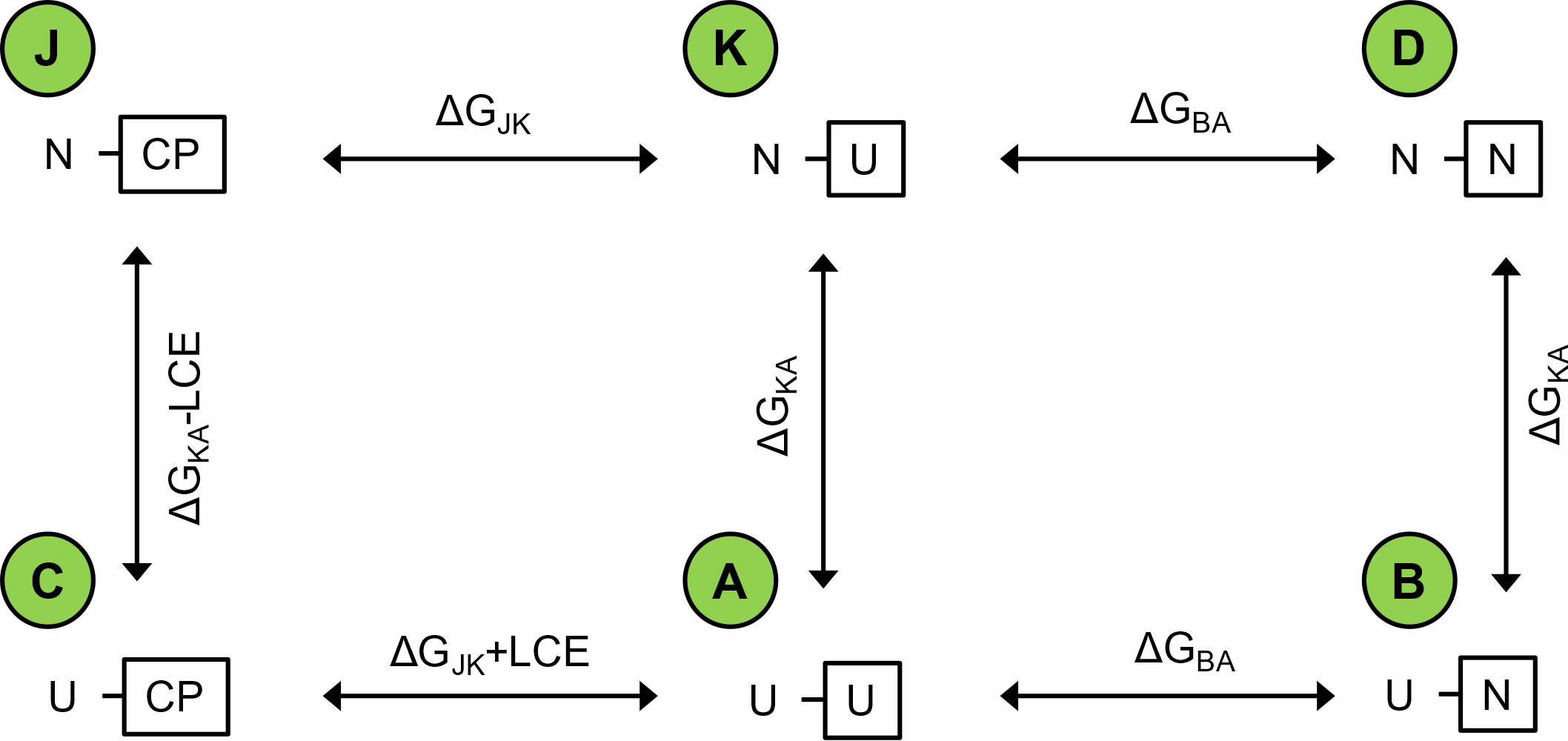
**

**Supplemental Figure S2.** **LCE-only switching simulation, supporting Fig. 2.** The LCE-only model consists of cpFKBP inserted into the CP-fold of Bn-AFF, yielding 6 states. Since FKBP is not present in this construct, the states are represented by a two-letter code corresponding to cpFKBP-Bn. The LCE penalty is imposed to any reaction in which the CP-fold folds when cpFKBP is unfolded. Conversely, an LCE “bonus” of equal magnitude and opposite sign is applied to any reaction in which cpFKBP folds when the CP-fold is already folded. Conservation of mass demands this condition, but it also makes intuitive sense: cpFKBP folding should be more favorable when its amino and carboxy termini are constrained to their native distance. Without rapamycin present, the molecules populate the states in which cpFKBP is unfolded (lower row), but unlike MEF they accumulate in **B** (OFF) even when the stabilities of the N-fold and CP-fold are identical (ΔΔG_N/CP_ = 0). This is because the CP-fold is penalized due to cpFKBP being unfolded, whereas the N-fold is not, and it explains why LCE resulted in lower background activity compared to MEF in Fig. 2. Adding rapamycin stabilizes any state in which cpFKBP is folded (upper row), causing the molecules to partition between **J** (ON) and **D** (OFF). Increasing ligand concentration stabilizes cpFKBP, but once cpFKBP is folded the LCE penalty imposed on folding of the CP-fold is removed, so the relative populations of **J** and **D** remain constant. The activity of the LCE-only switch at saturating [rapamycin] is therefore determined by the intrinsic stabilities of the N-fold and CP-folds, *i.e.*, ΔΔG_N/CP_. Changes in the distributions of populations as functions of [rapamycin] and ΔΔG_N/CP_ are shown in Fig. S4B. The following ΔG_unfold_ values (in units of kcal/mol) were used to generate the data in in Fig. 2 and Fig. S4B: ΔG_BA_ = 5; ΔG_KA_ = -2+ RTln(1+K_d_^-1^[rapamycin]); ΔG_JK_ was set to 5, 4, 3, and 2 to make ΔΔG_N/CP_ = 0, 1, 2, and 3; LCE = -2.


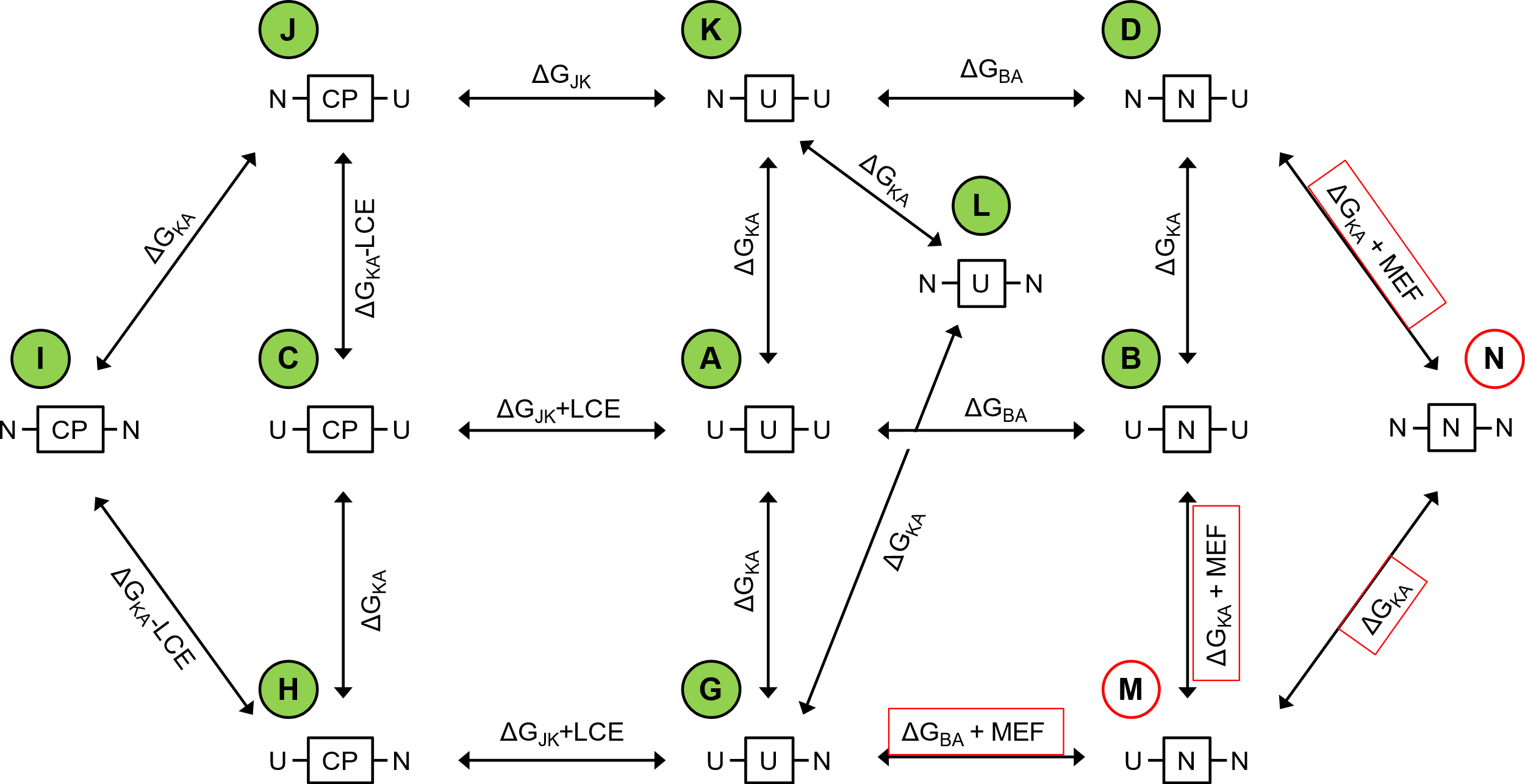


**Supplemental Figure S3.** **Simulation of combined MEF-LCE switching, supporting Fig. 2.** The switch constructed in this study consisted of FKBP inserted into the N-frame of Bn-AFF and cpFKBP inserted into the CP-frame. To generate the full model, one simply fuses the MEF-alone and LCE-alone schemes in Fig. S1 and Fig. S2 to yield the 12 states above. The transitions disallowed by the high MEF penalty are again boxed in red. At ΔΔG_N/CP_ = 0 and no rapamycin, the molecules populate states in which FKBP and cpFKBP are unfolded (middle row), biased to the N-fold (**B**) (OFF) as in Fig. S2 due to the LCE penalty. The low baseline activity of the LCE-only switch is therefore preserved. As rapamycin is added, states in which cpFKBP and FKBP are both folded (**I** and **N**) are preferentially stabilized. Since **N** is disallowed due to the MEF penalty, all molecules eventually accumulate in **I**, ensuring full turn-on of the switch. State **J** also accumulates at low [rapamycin] and is efficiently converted to **I** via the LCE effect, contributing to the enhanced performance of the dual switch at low ligand concentrations. Changes in the distributions of populations as functions of [rapamycin] and ΔΔG_N/CP_ are shown in Fig. S4C. The following ΔG_unfold_ values (in units of kcal/mol) were used to generate the data in in Fig. 2 and Fig. S4C: ΔG_BA_ = 5; ΔG_KA_ = -2+ RTln(1+K_d_^-1^[rapamycin]); ΔG_JK_ was set to 5, 4, 3, and 2 to make ΔΔG_N/CP_ = 0, 1, 2, and 3; LCE = -2.

**A**

**B**

**C**

ΔΔG=0

ΔΔG=1

ΔΔG=2

ΔΔG=3

**Supplemental Figure S4. Fractional changes of significantly populated states in the simulations, plotted as functions of [rapamycin] and ΔΔG_N/CP_, supporting Fig. 2.** The results of the simulations are shown for MEF-only switching **(A)**, LCE-only switching **(B)**, and combined MEF-LCE switching **(C)**. States with fractions of >0.02 at any [rapamycin] are shown and indicated in the inset; less populated states were omitted for clarity. Parameters used in the simulations are in the captions to Fig. S1, Fig. S2, and Fig. S3.

**A**

**B**

**Supplementary Figure S5: Raw data from urea denaturation experiments, supporting Fig. 3.** Denaturation curves of the N-fold analog **(A)** and the CP-fold analog **(B)** in the absence (black) or presence (blue) of 20 µM FK506 were performed at 4 °C in 50 mM HEPES (pH 7.0), 100 mM NaCl, and 0.01% Tween-20. The fitted baselines were used to calculate fraction unfolded in Fig. 3. Data are plotted as mean ± s.d. of 3 technical repeats. Lines are the fits for 2-state linear extrapolation equation. The fitted parameters are reported in Table S1.


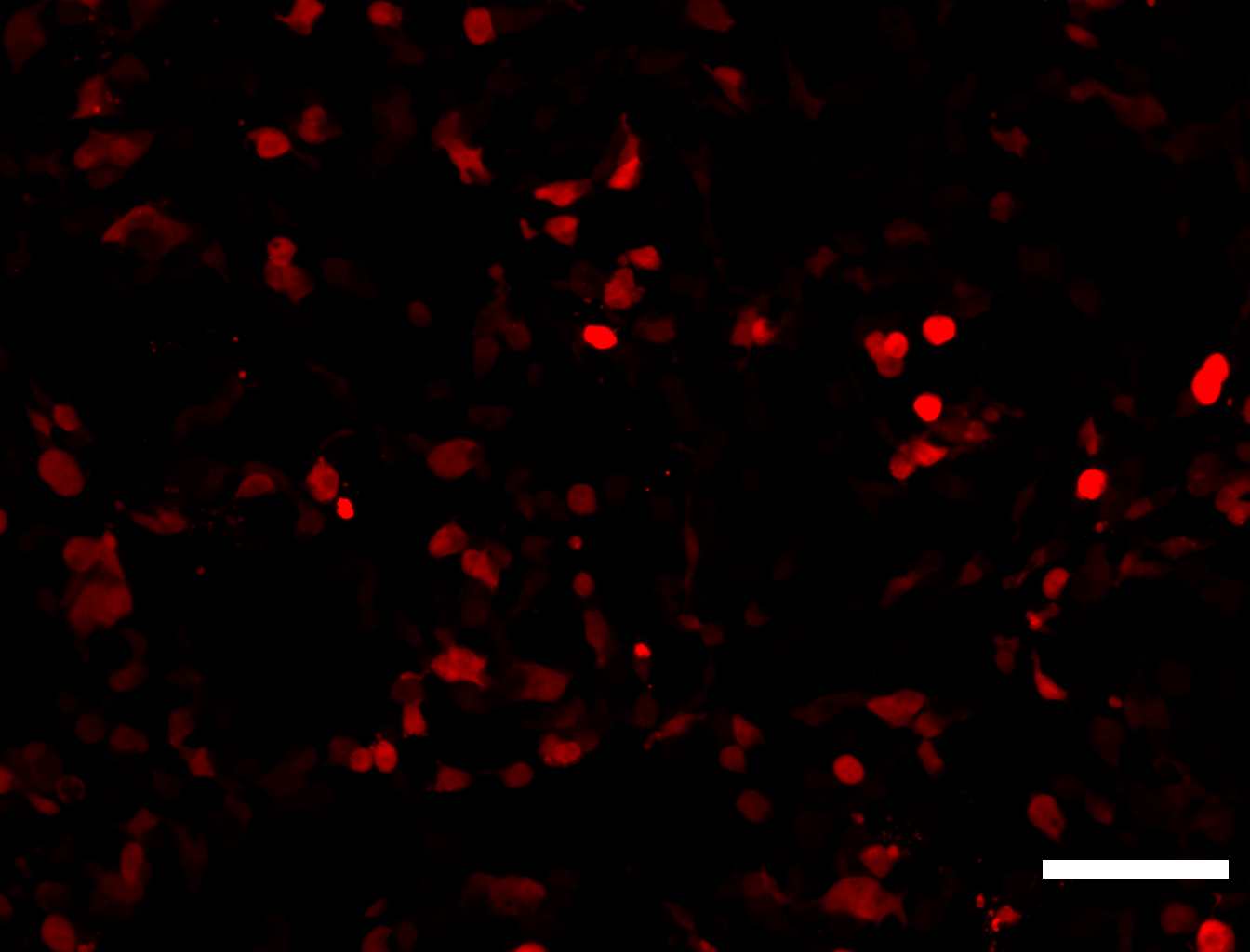

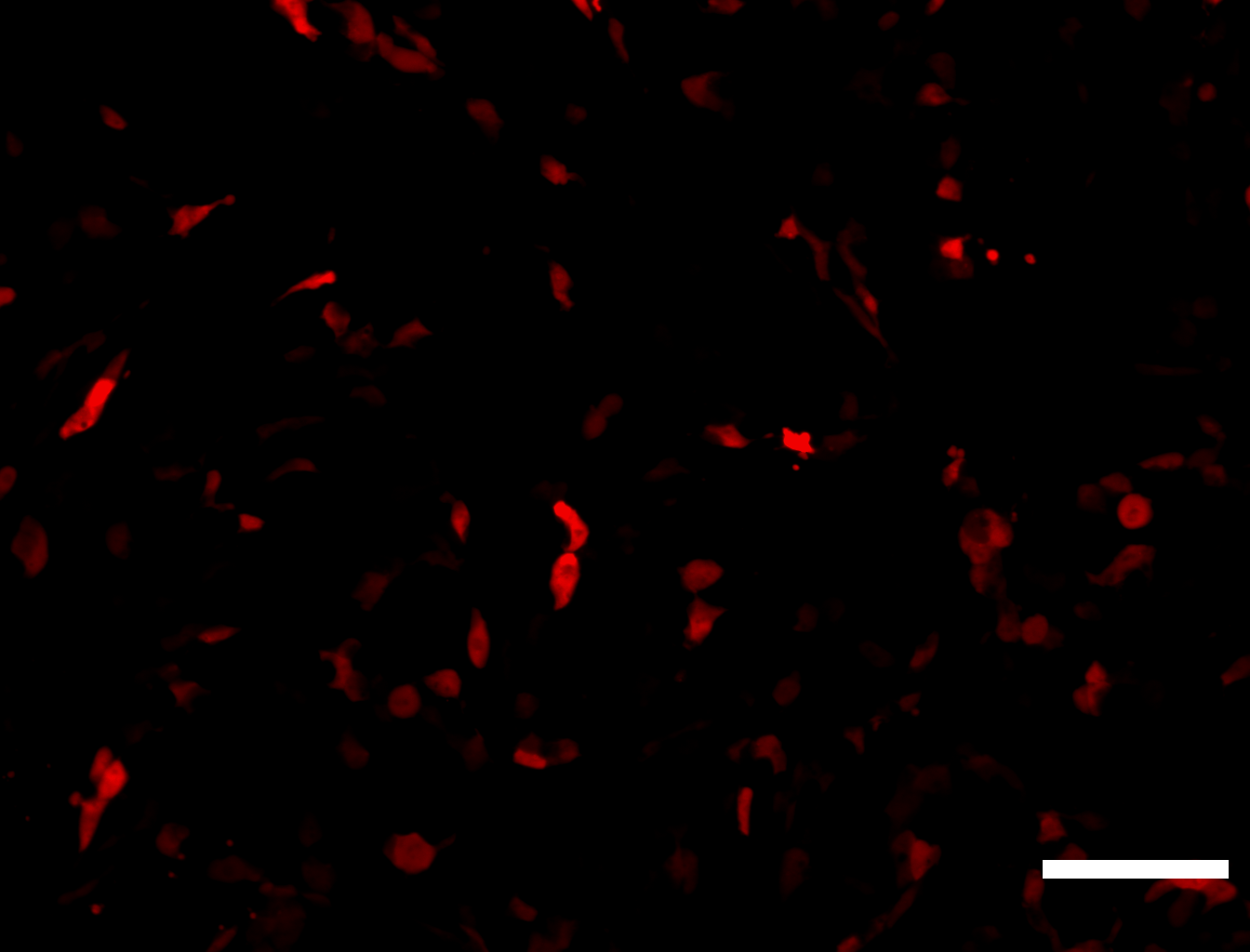

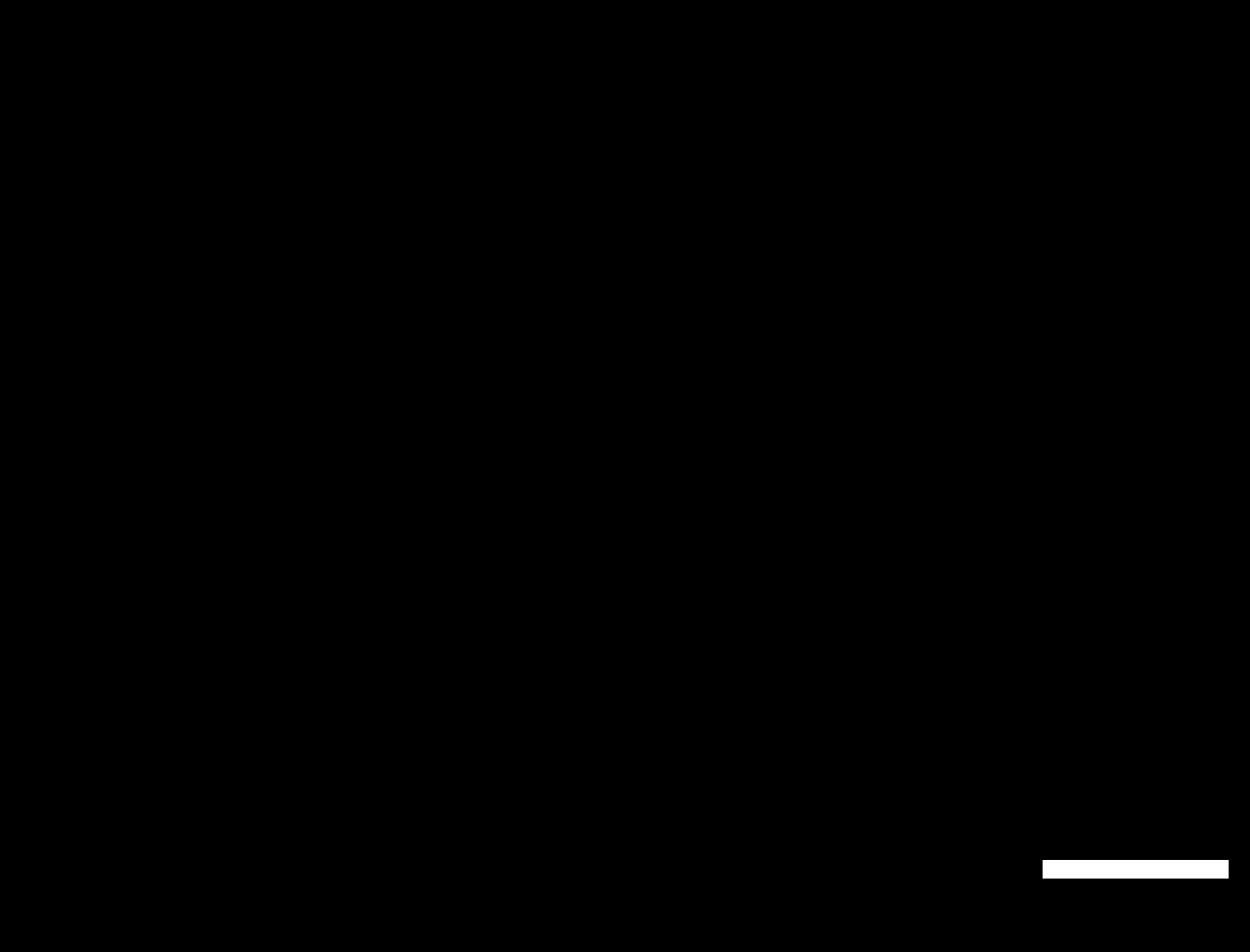

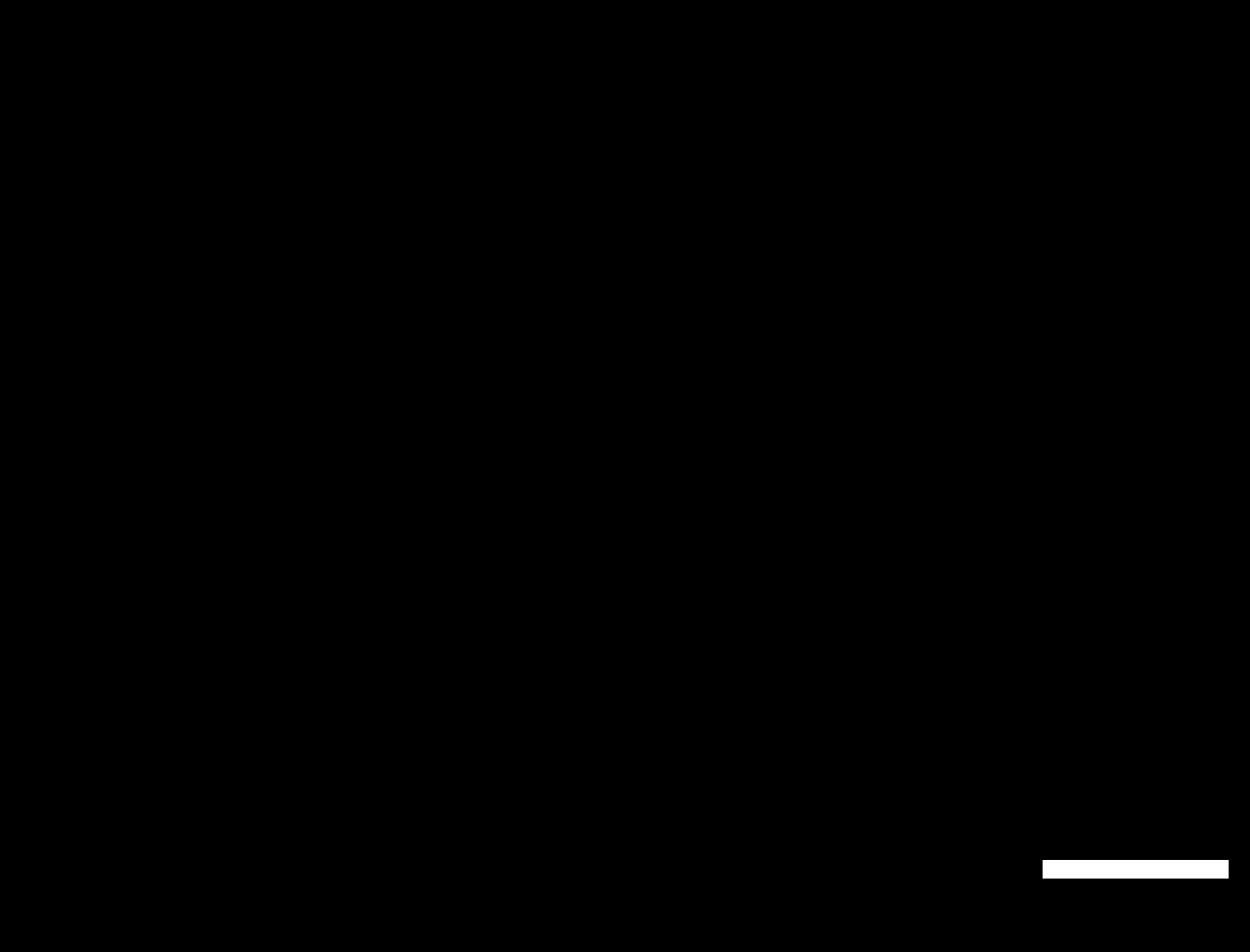

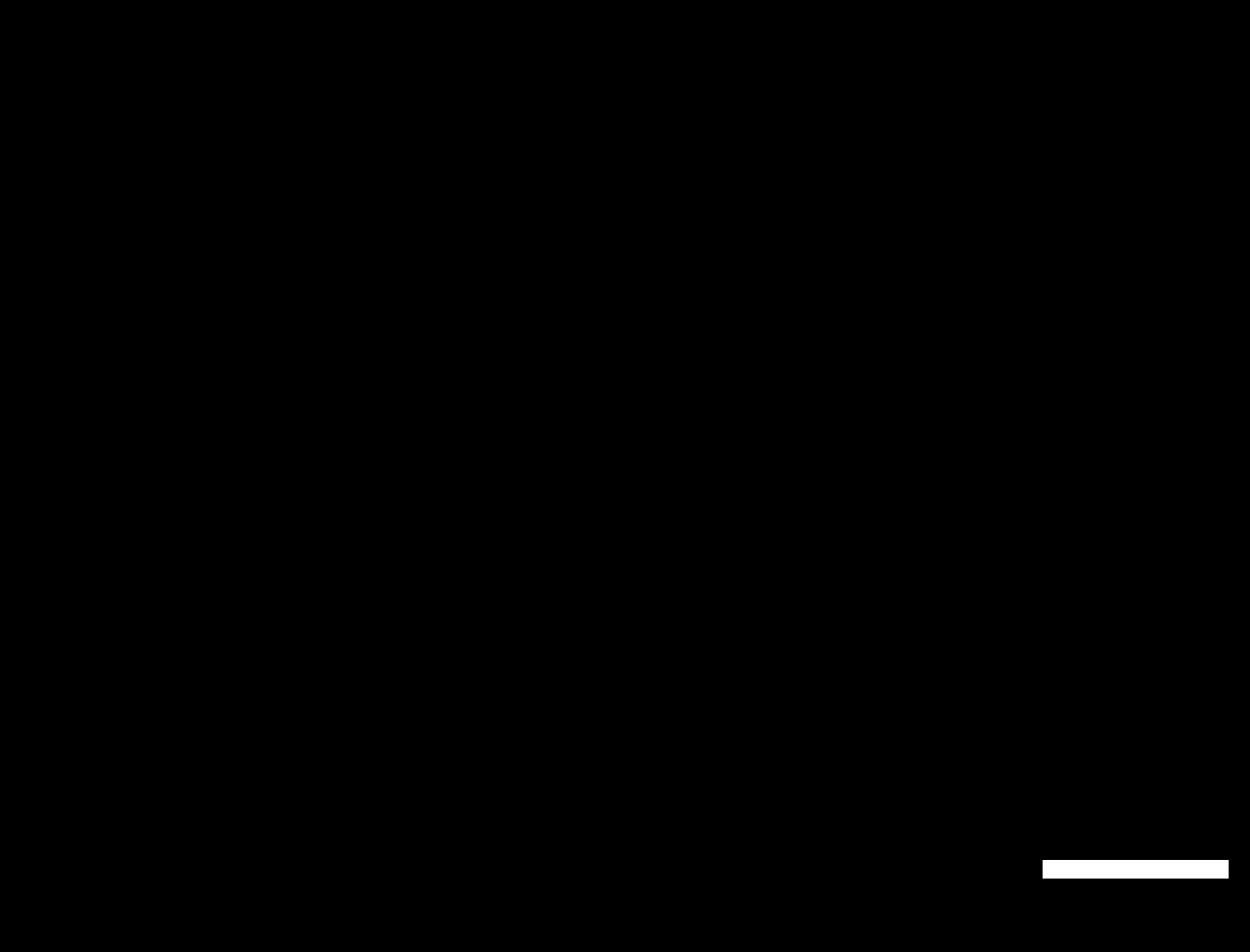

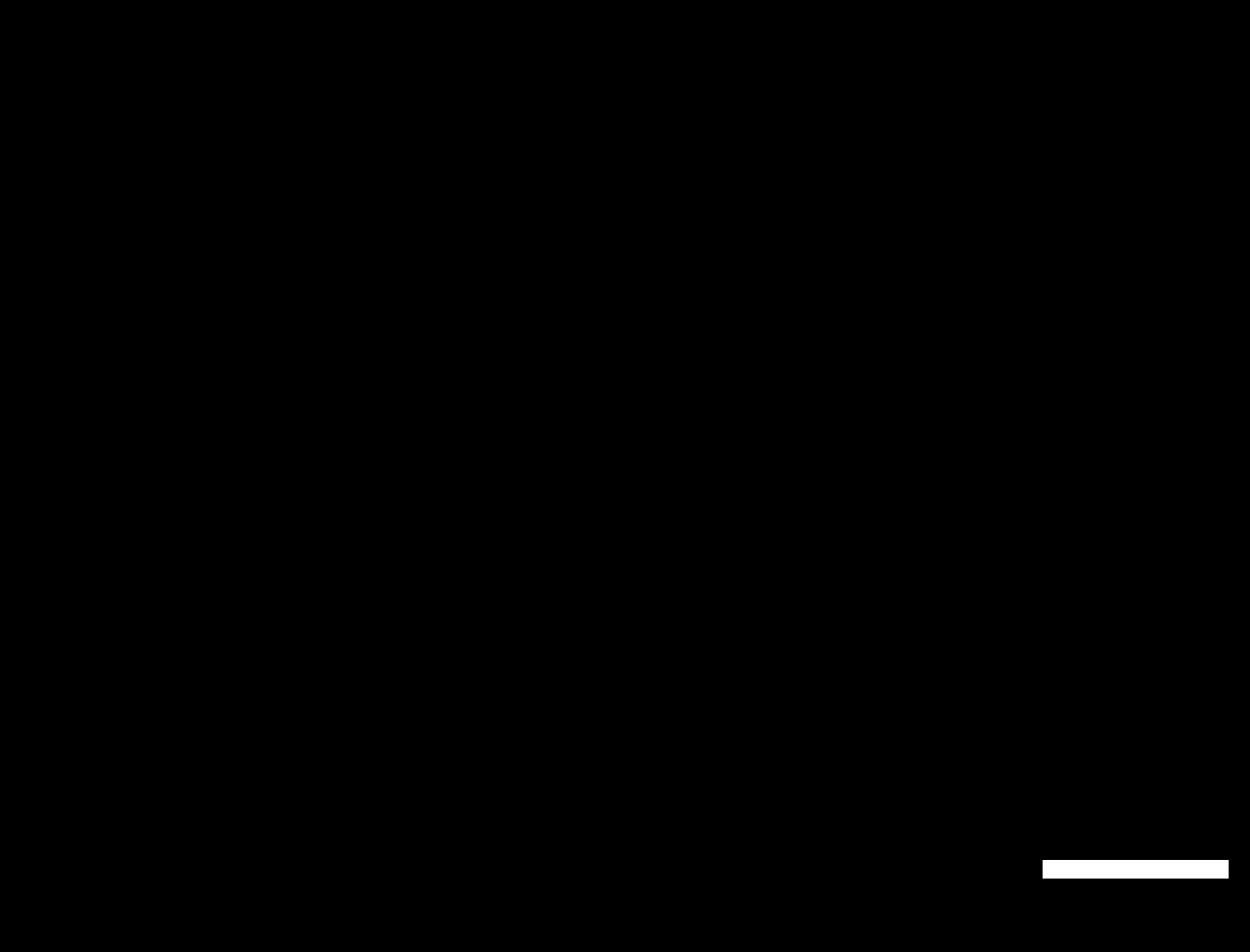

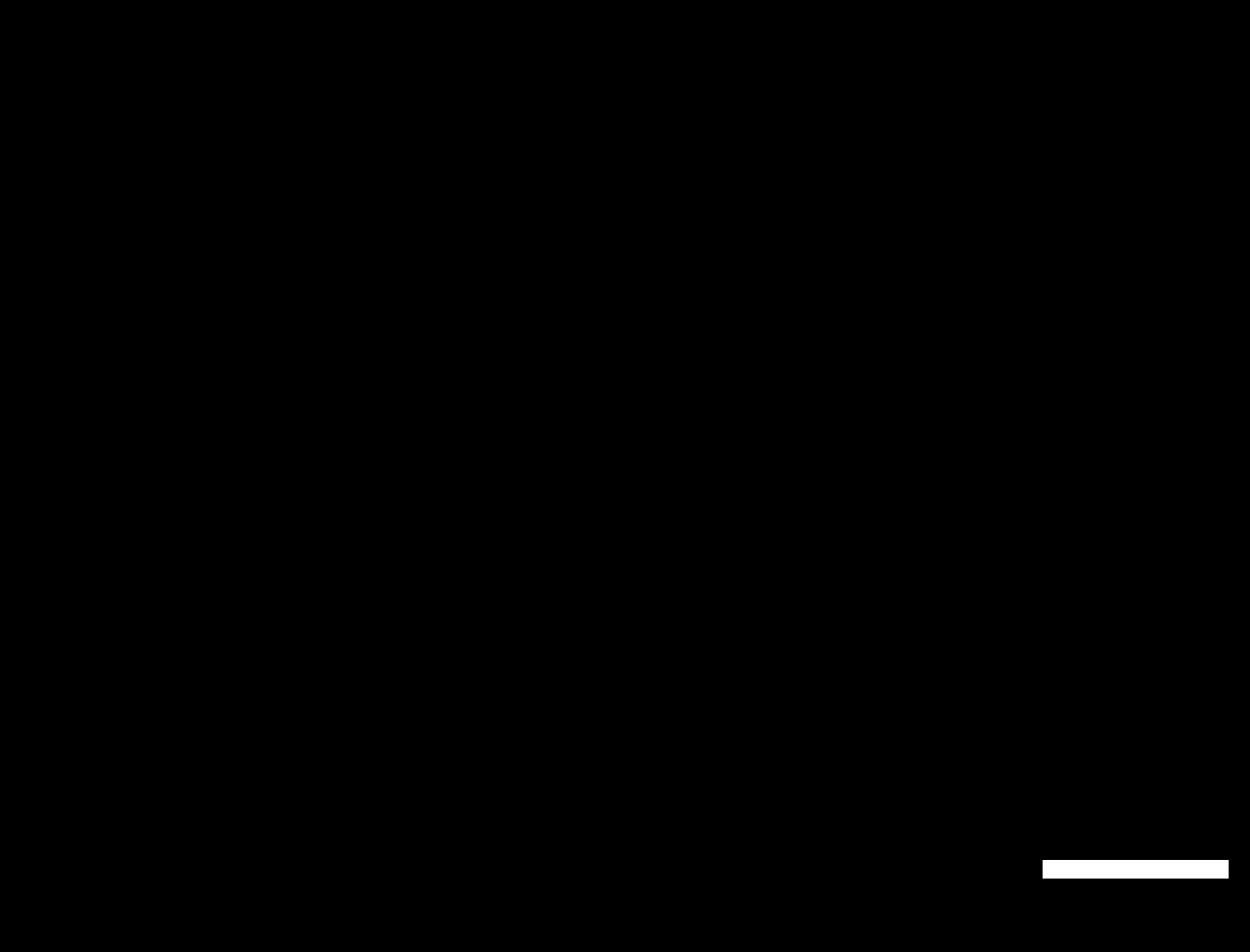

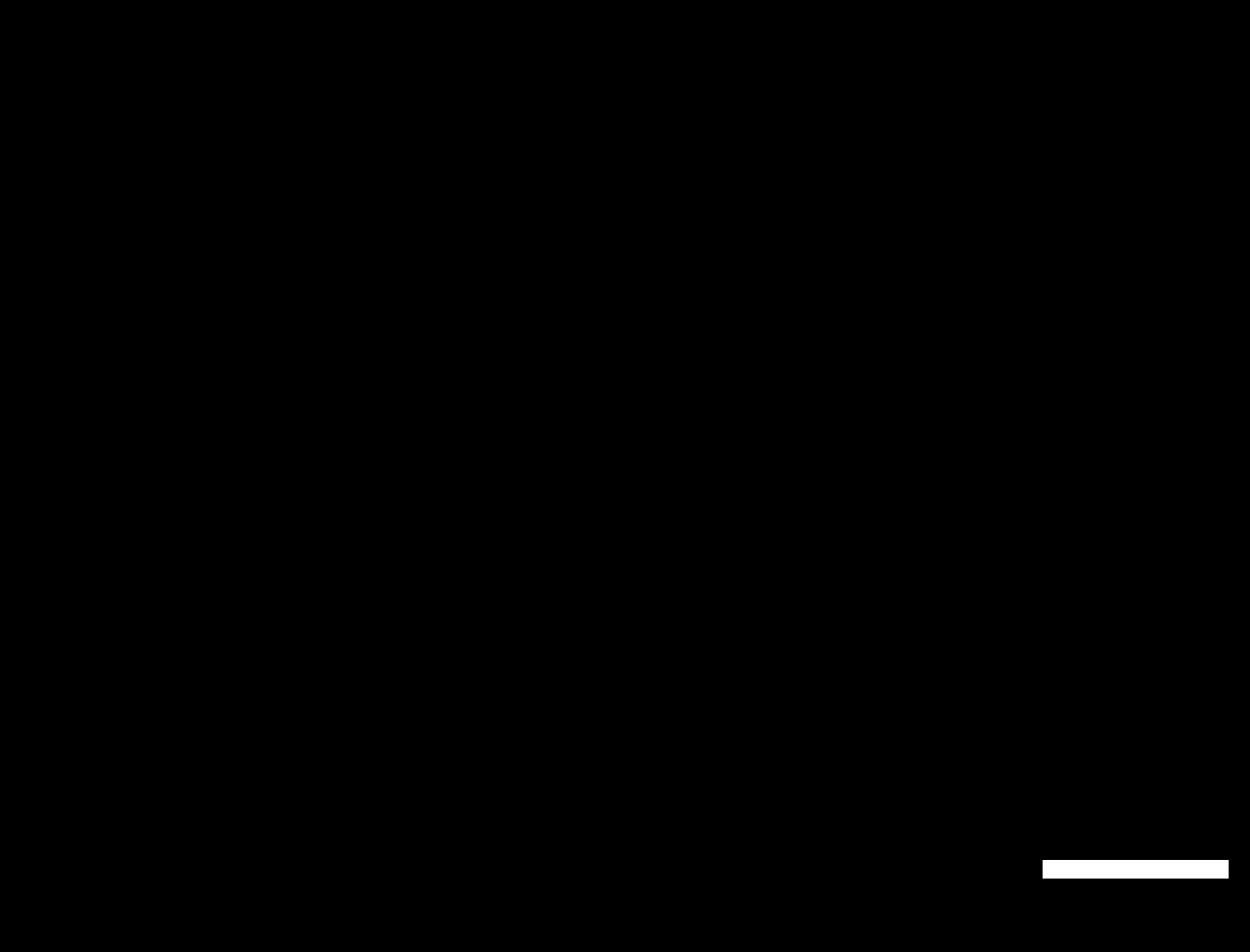

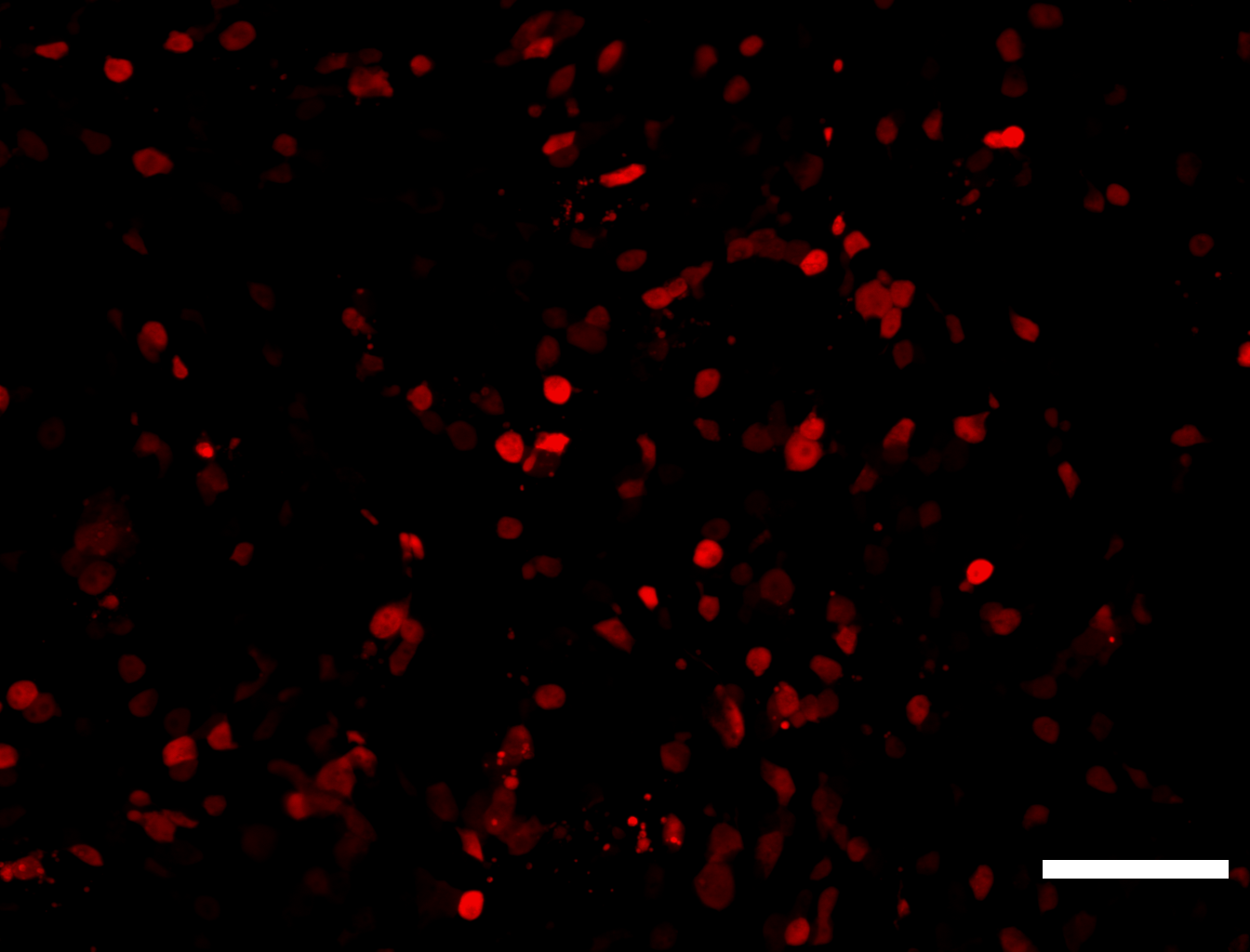

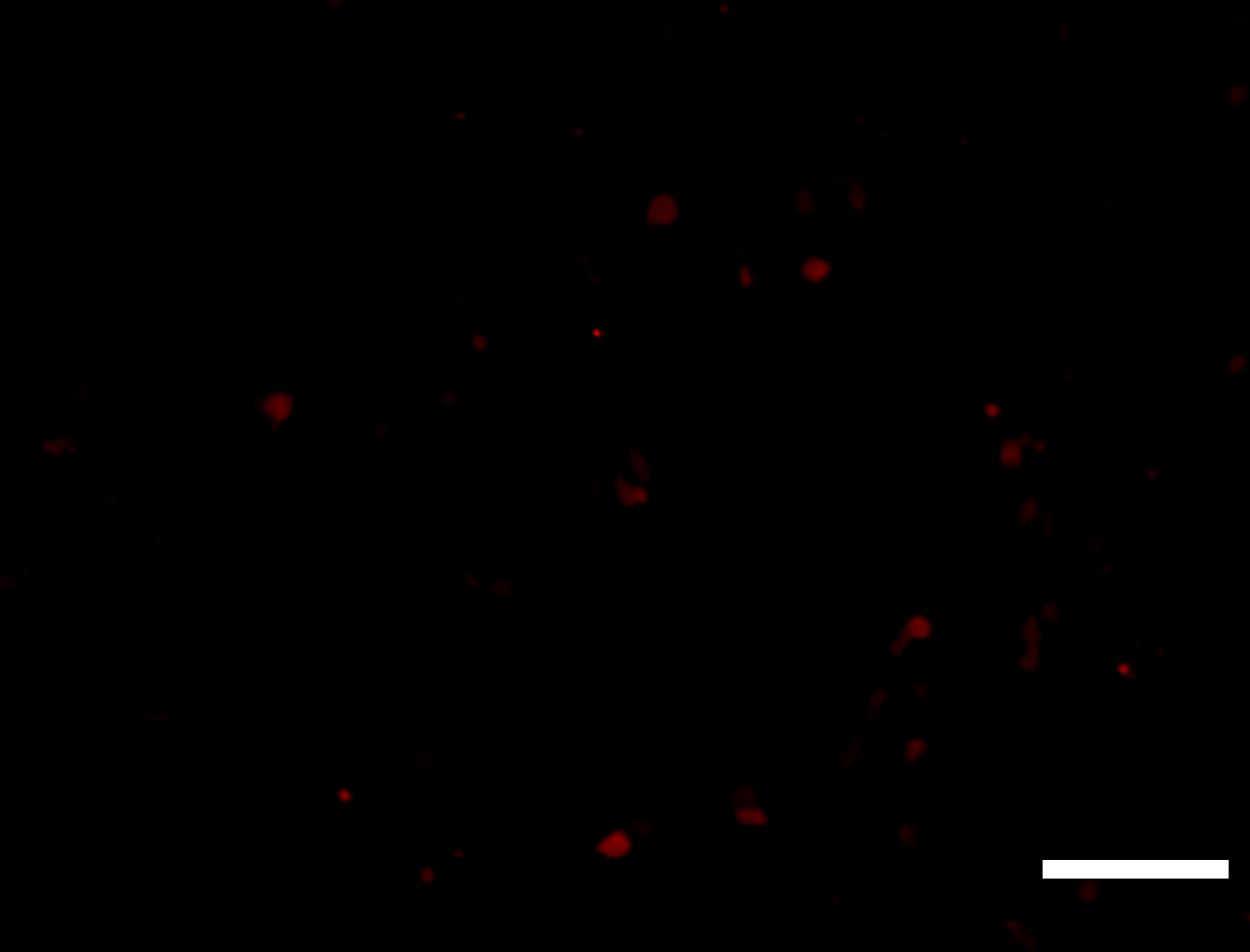

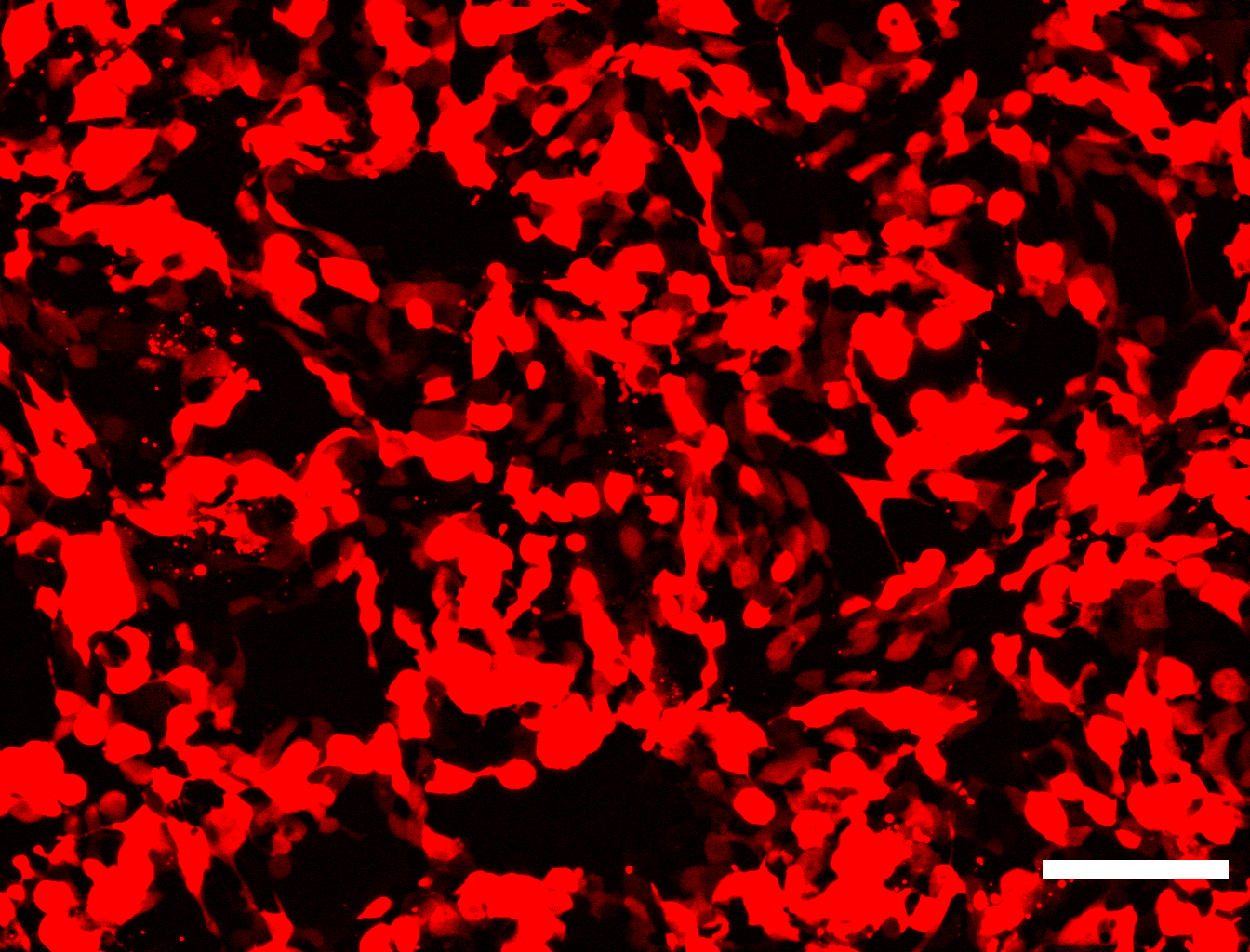

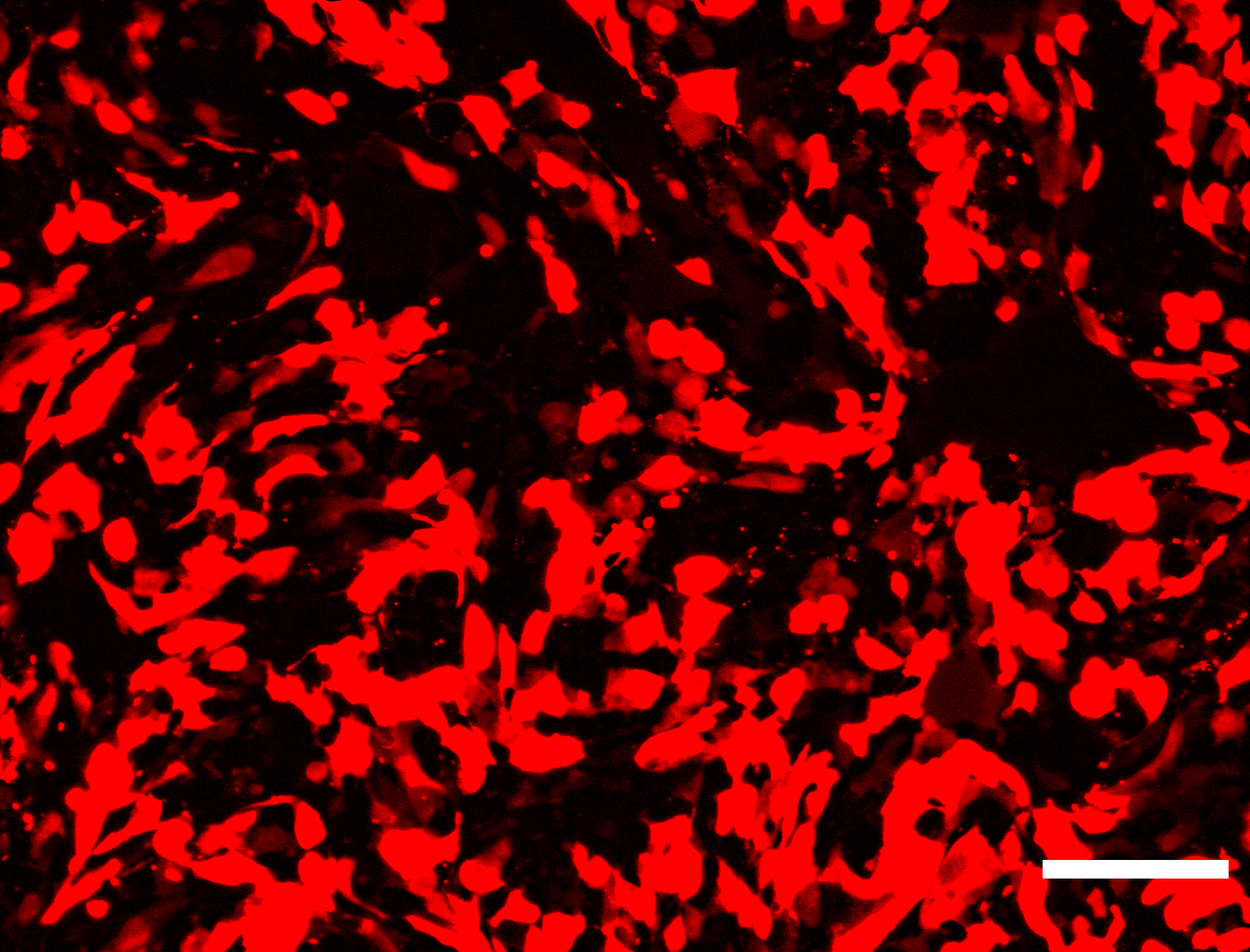

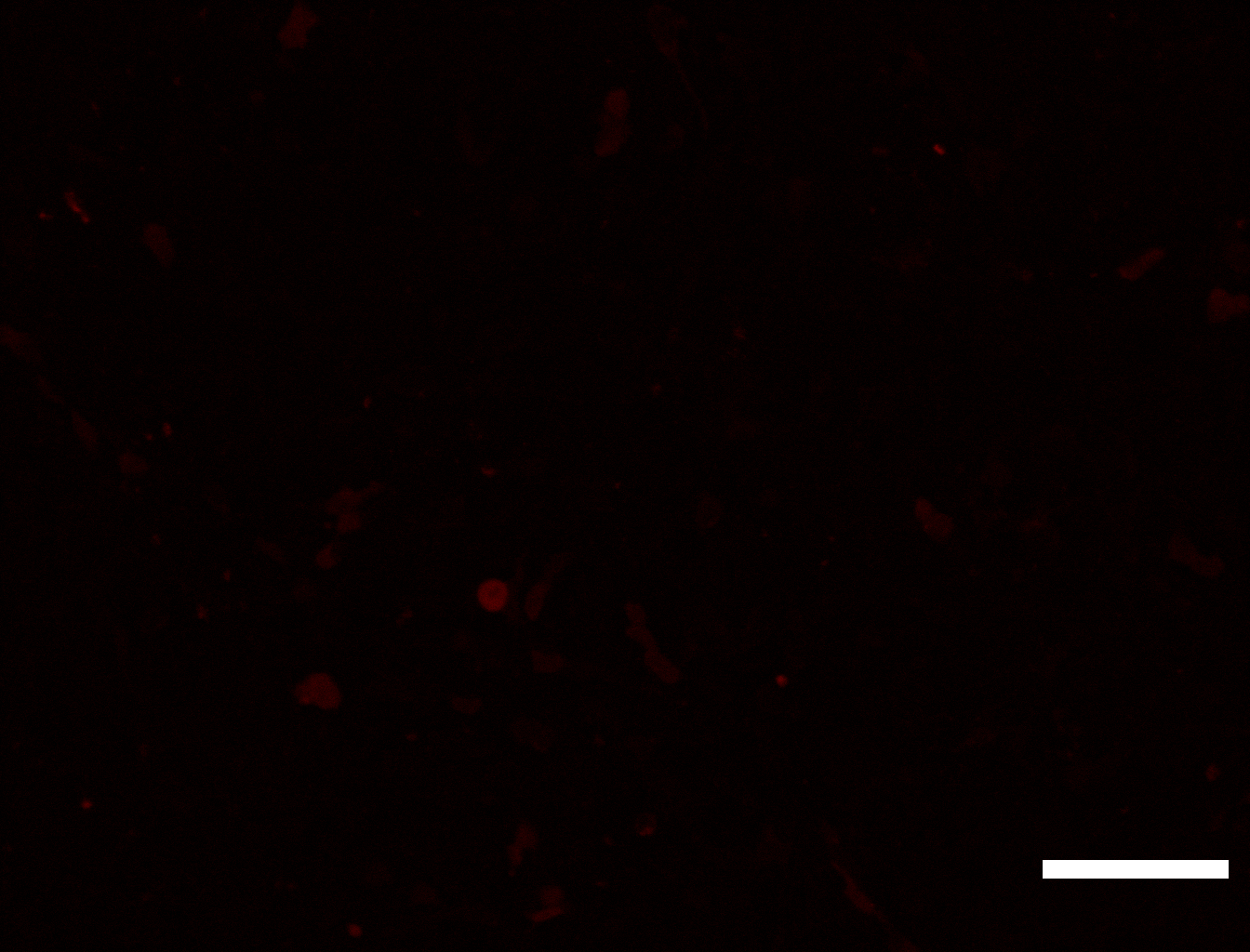

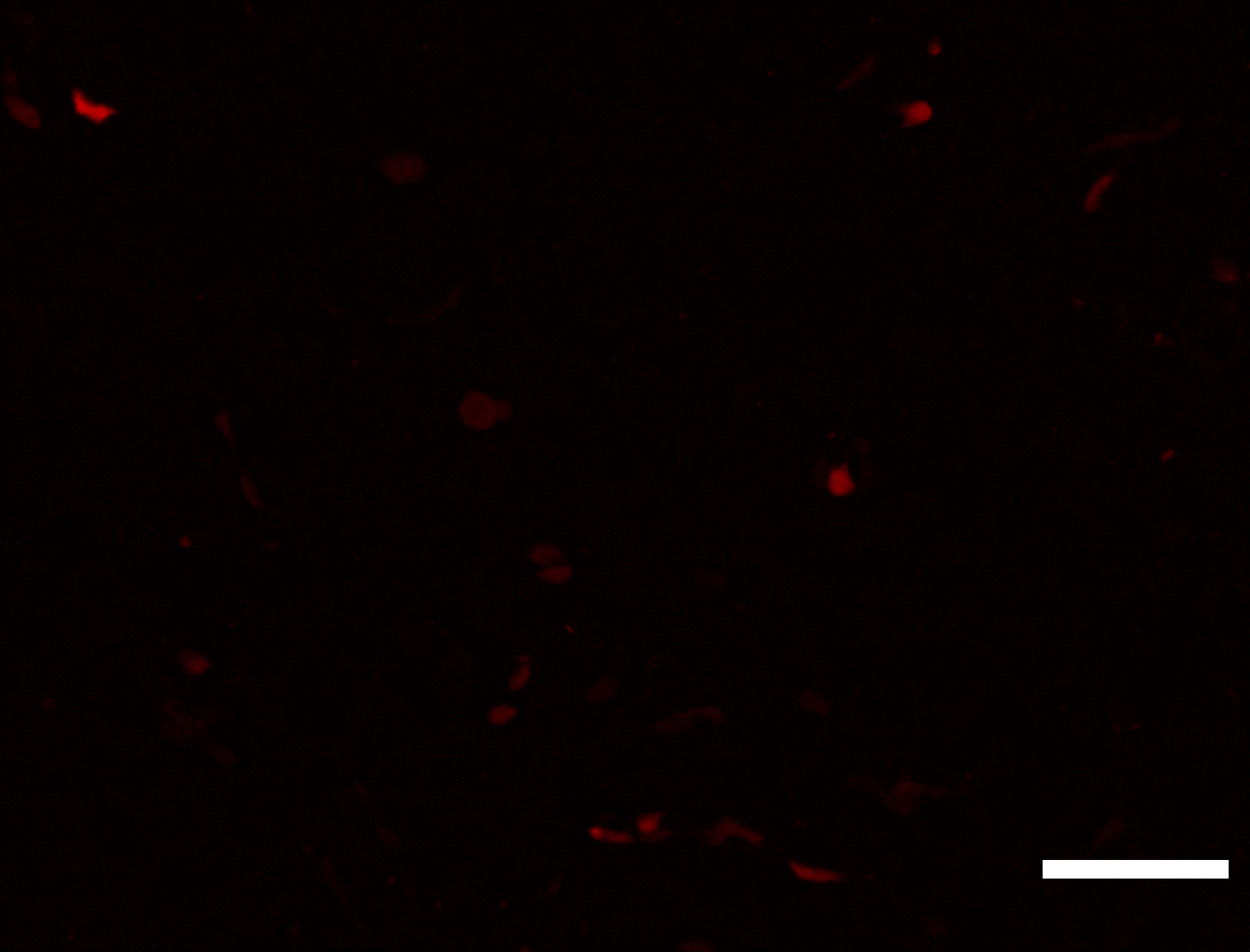

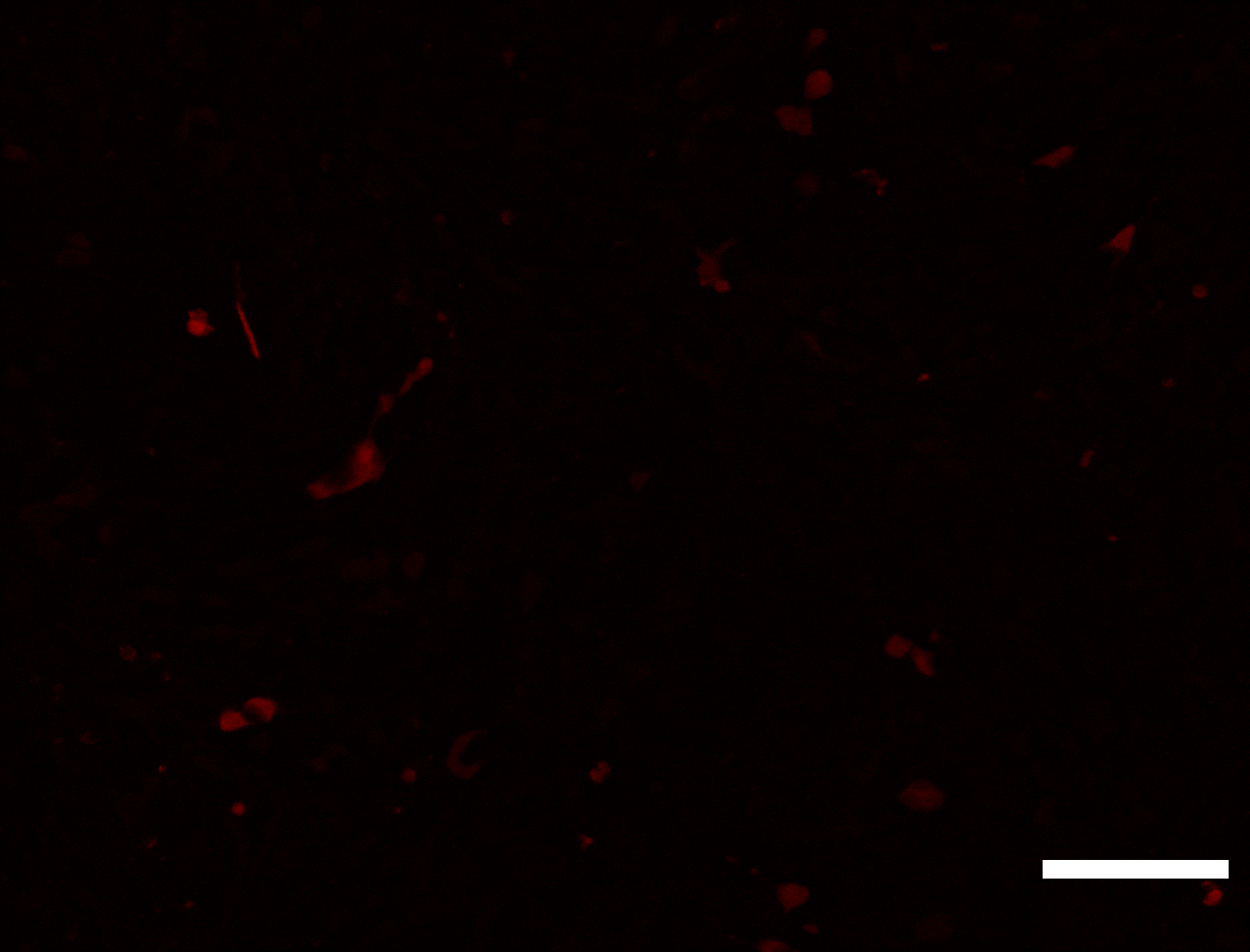

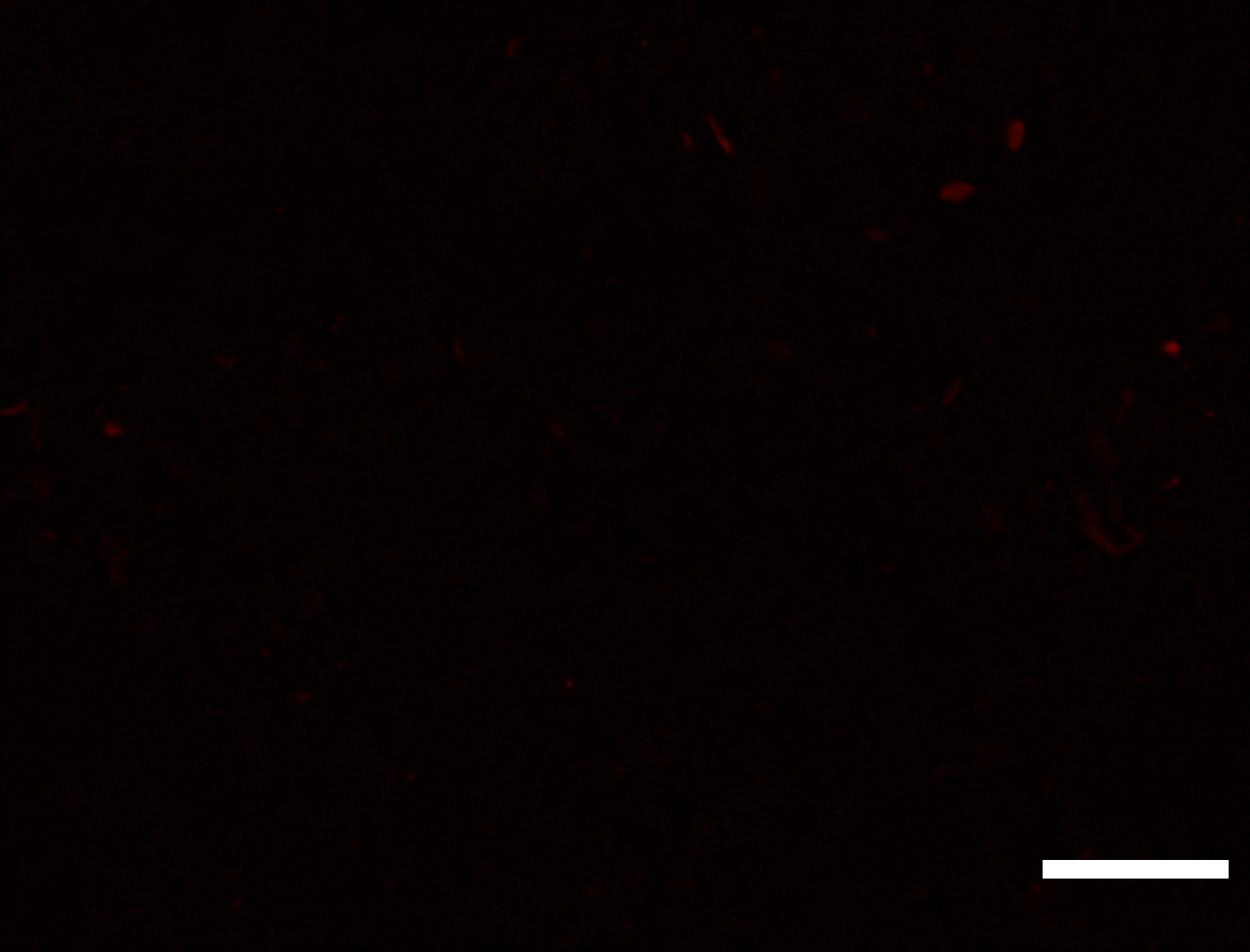

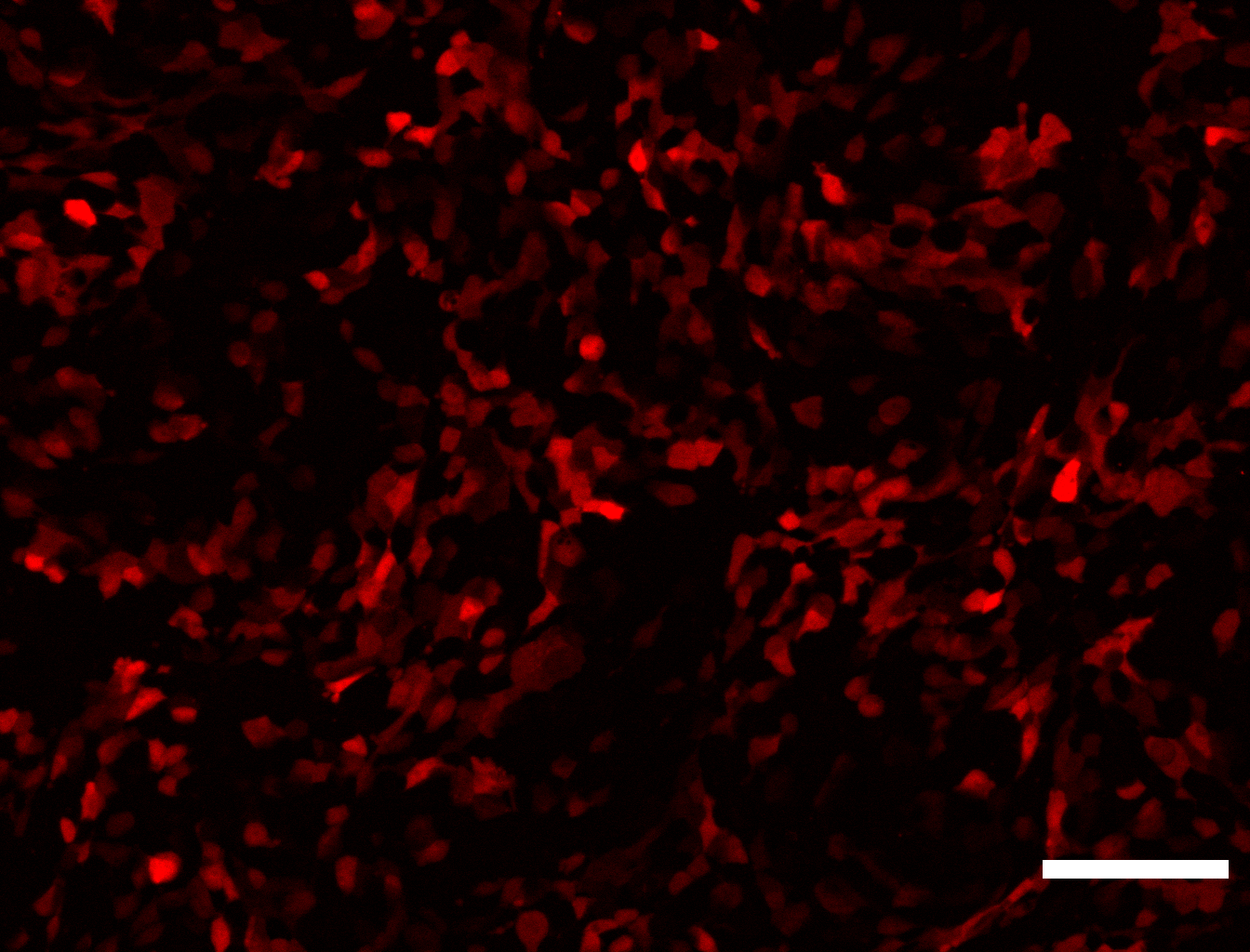

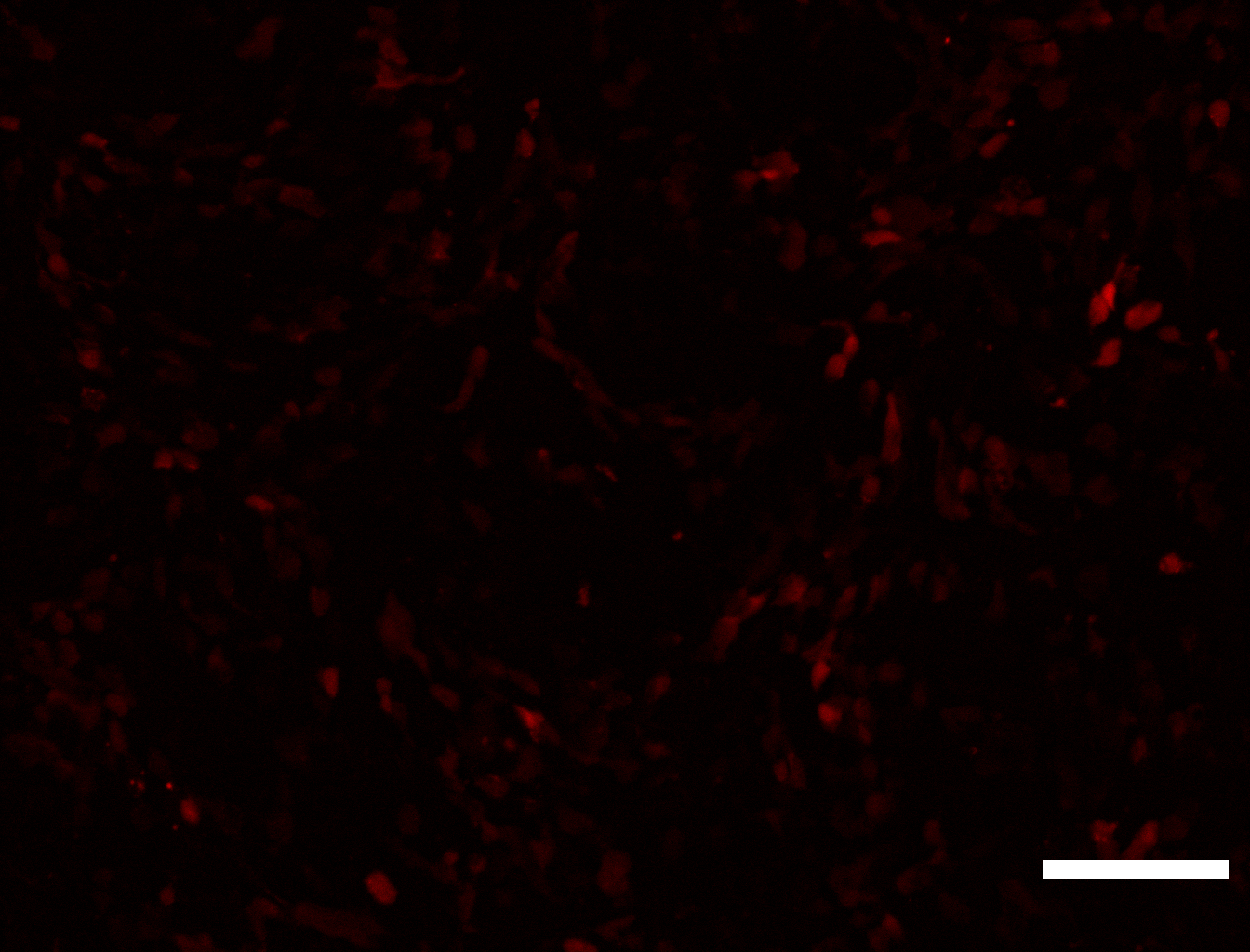

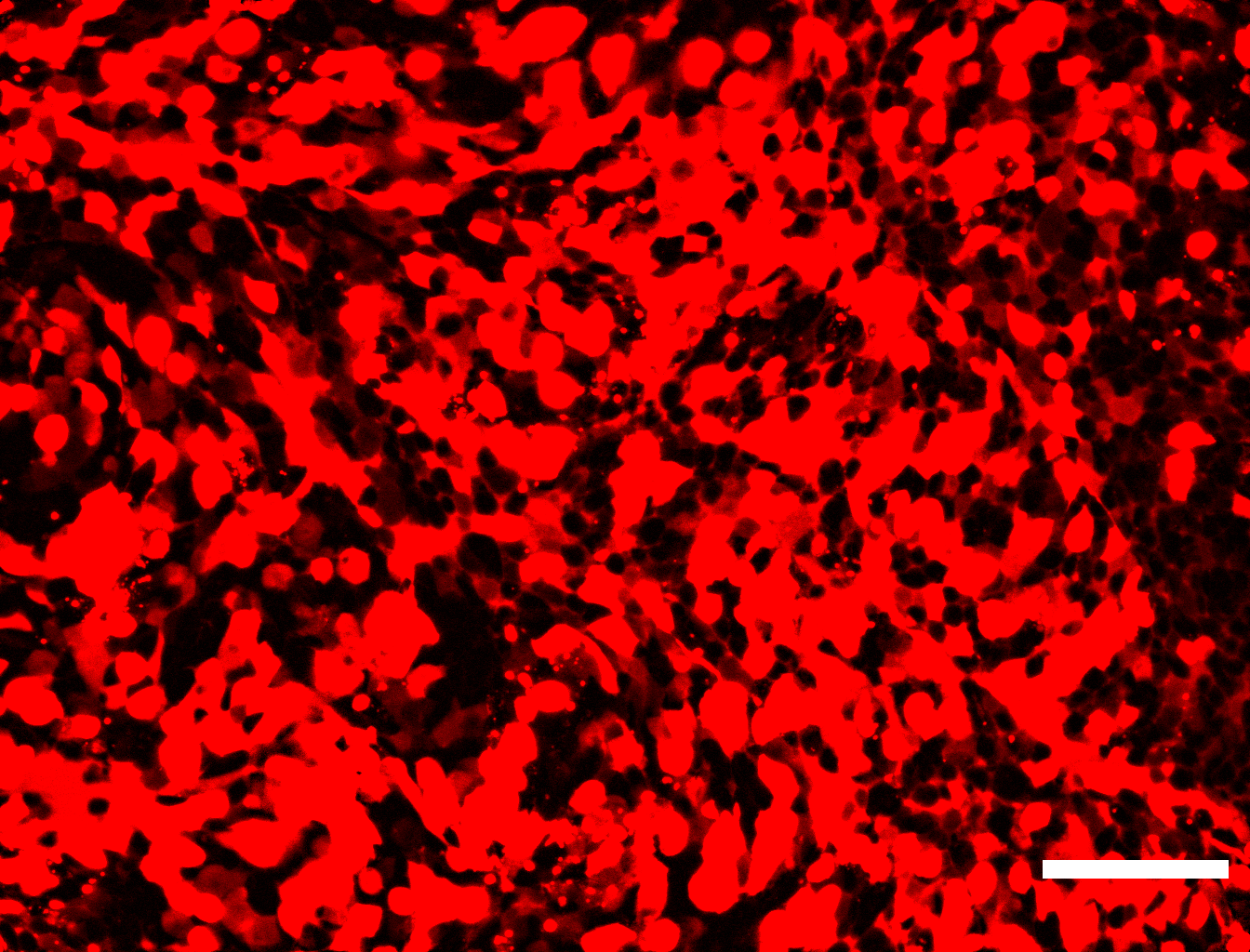

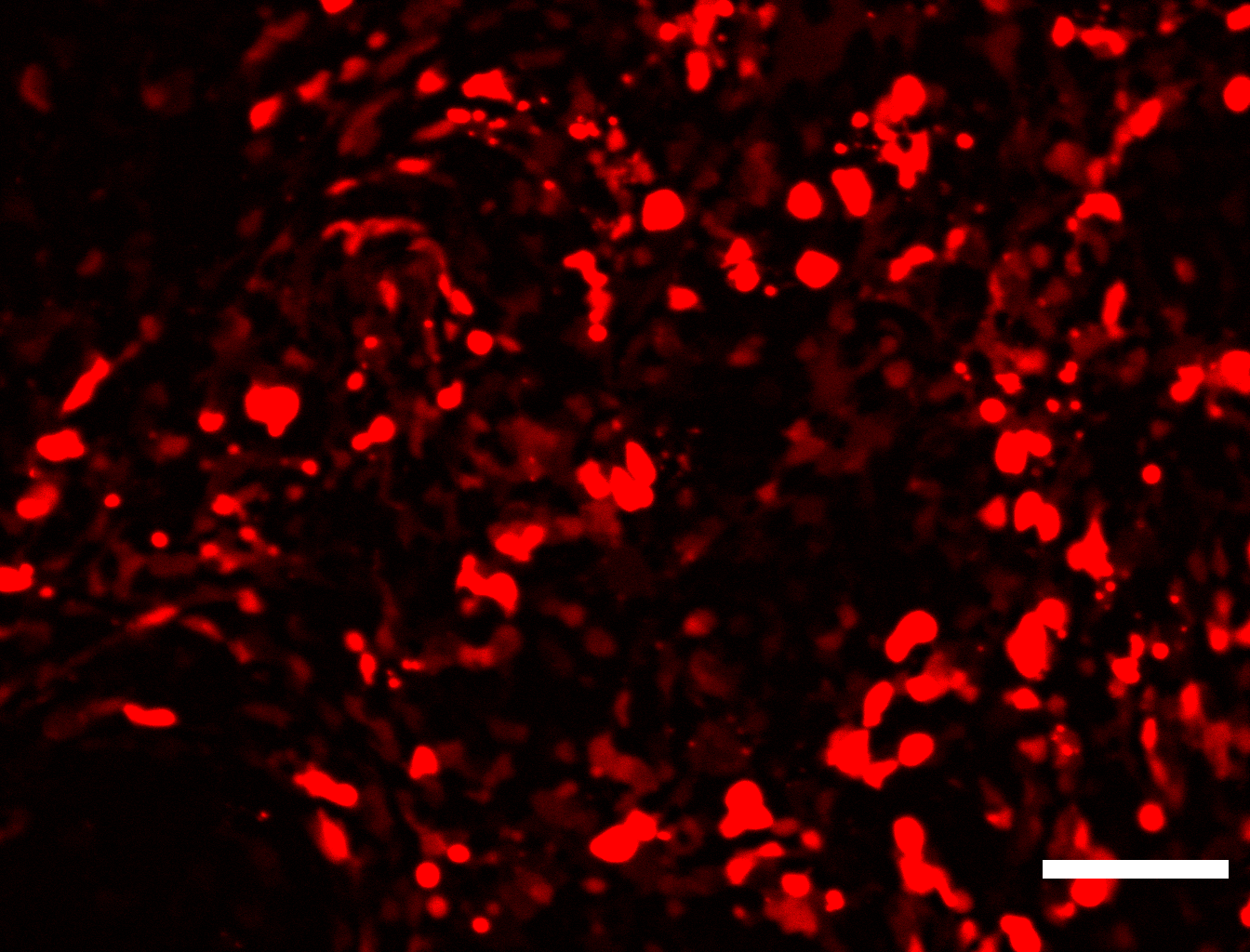


H102*A

WT

I96*V

I96*A

I96*G

Vehicle

Rapamycin

Vehicle

Rapamycin

Low contrast

High contrast

**Supplementary Figure S6: Thermodynamic tuning of Bn-AFF in HEK293T cells, supporting Fig. 4.** Four plasmids encoding Bn-AFF variants with increasing ΔΔG_N/CP_ values increasing in the order WT, I96*V, I96*A, I96*G, and a null-activity control plasmid H102*A, were transfected along with a mCherry-PEST reporter plasmid. The mCherry fluorescence channel is shown at low contrast (left) and high contrast (right). As predicted by the model in Supplementary Note 1, background RNase activity of the switches decreased as ΔΔG_N/CP_ increased due to destabilization of the CP-fold. This effect can be seen by the trend of increasing mCherry fluorescence of WT, I96*V, I96*A, and I96*G cells treated with DMSO vehicle, especially at high contrast. Rapamycin treatment (2.5 µM) activated the I96*A and I96*G switches as evidenced by the decreased fluorescence. No effect of rapamycin was observed for WT and I96*V due to their high background activities.

**A**

**B**

**C**

**Supplementary Figure S7: Thermal denaturation curves for the CP-fold analogs of I96*G and I96*A mutants, supporting Fig. 6. (A)** I96*G showed a significantly reduced thermal stability compared to I96*A in 20 mM phosphate (pH 7.0), 100 mM NaCl, and 20 μM FK506. Data are plotted as mean ± s.d. of three technical repeats. Lines are meant to guide the eye only. T_m_ values, calculated by fitting the derivative of the melting curves for each replicate (n = 3) to a gaussian function, were 23.2 ± 0.8 ^o^C for the I96*G mutant **(B)** and 30.5 ± 0.2 ^o^C for the I96*A mutant **(C).**

| **Frame** |  | **ΔG_unfolding_ (kcal/mol)** | ***m* (kcal/mol/M Urea)** | **C_m_ (M Urea)** |
| --- | --- | --- | --- | --- |
| CP | (-) FK506 | 3.3 ± 0.1 | 1.94 ± 0.03 | 1.76 ± 0.01 |
|  | (+) FK506 | 5.7 ± 0.7 | 3.0± 0.4 | 1.99 ± 0.06 |
| Native | (-) FK506 | 7.6 ± 0.7 | 2.3 ± 0.2 | 3.2 ± 0. 1 |
|  | (+) FK506 | 3.2 ± 0.1 | 1.1± 0.2 | 3.1 ± 0.2 |

**Supplementary Table 1: Thermodynamic parameters from urea denaturation experiments, supporting Fig. 3.** Parameters were obtained by fitting the data to the linear extrapolation equation. Reported values are mean ± s.d. (3 technical repeats).
